# Innervation Drives Postembryonic Expansion of the Zebrafish Anterior Lateral Line System

**DOI:** 10.1002/cne.70132

**Published:** 2026-01-22

**Authors:** Theresa J. Christiansen, Vishruth Venkataraman, Victoria E. Prince

**Affiliations:** ^1^ Department of Organismal Biology & Anatomy The University of Chicago Chicago Illinois USA

**Keywords:** anterior lateral line, canal neuromast, lateral line ganglia, lateral line nerve, superficial neuromast, zebrafish

## Abstract

The lateral line system is an essential sensory modality used by fishes and aquatic amphibians to sense hydrodynamic information. The system comprises distributed sense organs called neuromasts and their afferent nerves, organized into anterior lateral lines around the eye and jaw and posterior lateral lines (LL) on the trunk. At postembryonic stages, early forming neuromasts expand in size and sink into bony canals, while late‐forming superficial neuromasts are added as the fish grows. Unlike the well‐studied zebrafish posterior LL, details of anterior LL postembryonic development remain unknown. Here, we have characterized developmental mechanisms and innervation patterns driving expansion of the zebrafish anterior LL. Using tissue‐clearing to observe neuromast and nerve markers through ontogeny, we demonstrate continuous neuromast addition in the anterior LL, with peak rates at larval stages of 7–10 mm standard length (SL). Lines of superficial neuromasts form parallel to existing lines of presumptive canal neuromasts as late as 7 mm SL, with new neuromasts added through migration of new primordia, budding, intercalation, and a novel “hybrid‐origin” mechanism. Despite some canal lines being innervated by the anterodorsal ganglion, all superficial lines are innervated by the anteroventral ganglion. Anterior LL ganglion ablation reveals that denervation abrogates superficial neuromast formation—including via the hybrid‐origin mechanism—and reduces growth of canal neuromasts. While the anterior and posterior LL use disparate developmental mechanisms, innervation is critical to the expansion of both. Our findings reveal a “developmental switch” at 7 mm SL, when innervation becomes necessary for a secondary phase of anterior LL development.

## Graphical abstract

A dramatic expansion of the anterior lateral line system occurs during zebrafish ontogeny, largely driven by the addition of superficial neuromasts. Here, we describe a new hybrid‐origin mechanism of neuromast addition and uncover a larval‐stage developmental switch to an innervation‐dependent phase of expansion.

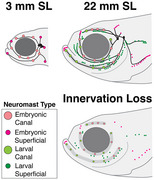

## 1 Introduction

The lateral line (LL) is a sensory system found in fishes and amphibians that allows them to sense hydrodynamic information from their aquatic environment (Dijkgraaf 1963; Mogdans 2019). The system is essential for complex behaviors such as schooling, hunting, and predator evasion (Mogdans 2019; Mogdans and Bleckmann 2012). The LL comprises distributed lines of mechanosensory organs called neuromasts innervated by afferent nerves, which relay sensory input to the LL ganglia and then to the medial octavolateral nucleus of the hindbrain (Raible and Kruse 2000). Each neuromast comprises a cluster of sensory hair cells and their surrounding support cells, with the hair cells projecting sensory cilia into an overlying jelly‐like cupula that interacts directly with the water stream. These neuromasts are found within the epithelial linings of fluid‐filled, bony canals—open to the water via pores—or located superficially in the epidermis (reviewed by Venkataraman, Lopez, et al. 2025). Adult patterns of neuromast distribution are complex and vary widely, even between closely related species (Webb 2014). These variations may be adaptations to varied flow environments (Mogdans 2019).

The neuromast receptors and their associated nerves develop from ectodermal thickenings, the cranial placodes. While cranial placodes give rise to a variety of sensory systems, including the olfactory, optic, and auditory capsules, a separate series of placodes gives rise to LL receptors and their nerves (Baker and Bronner‐Fraser 2001). The placodes lying just anterior to the otic vesicle produce the anterior LL system that populates the head, while placodes just posterior to the otic vesicle produce the posterior LL system that populates the trunk (Piotrowski and Baker 2014). The ontogeny of neuromast patterning has been described for several teleost species, including both the anterior and posterior LL systems of the cichlid genera *Tramitichromis* and *Aulanocara* (Becker et al. 2016; Webb 1990), the anterior LL of the brook trout (Jones et al. 2024) and the goby genus *Elacatinus* (Nickles et al. 2020), the posterior LL of medaka (Seleit et al. 2017), and the anterior and posterior LL systems of the zebrafish (Gompel et al. 2001; Ledent 2002; Sapède et al. 2002; Webb and Shirey 2003).

**FIGURE 1:**
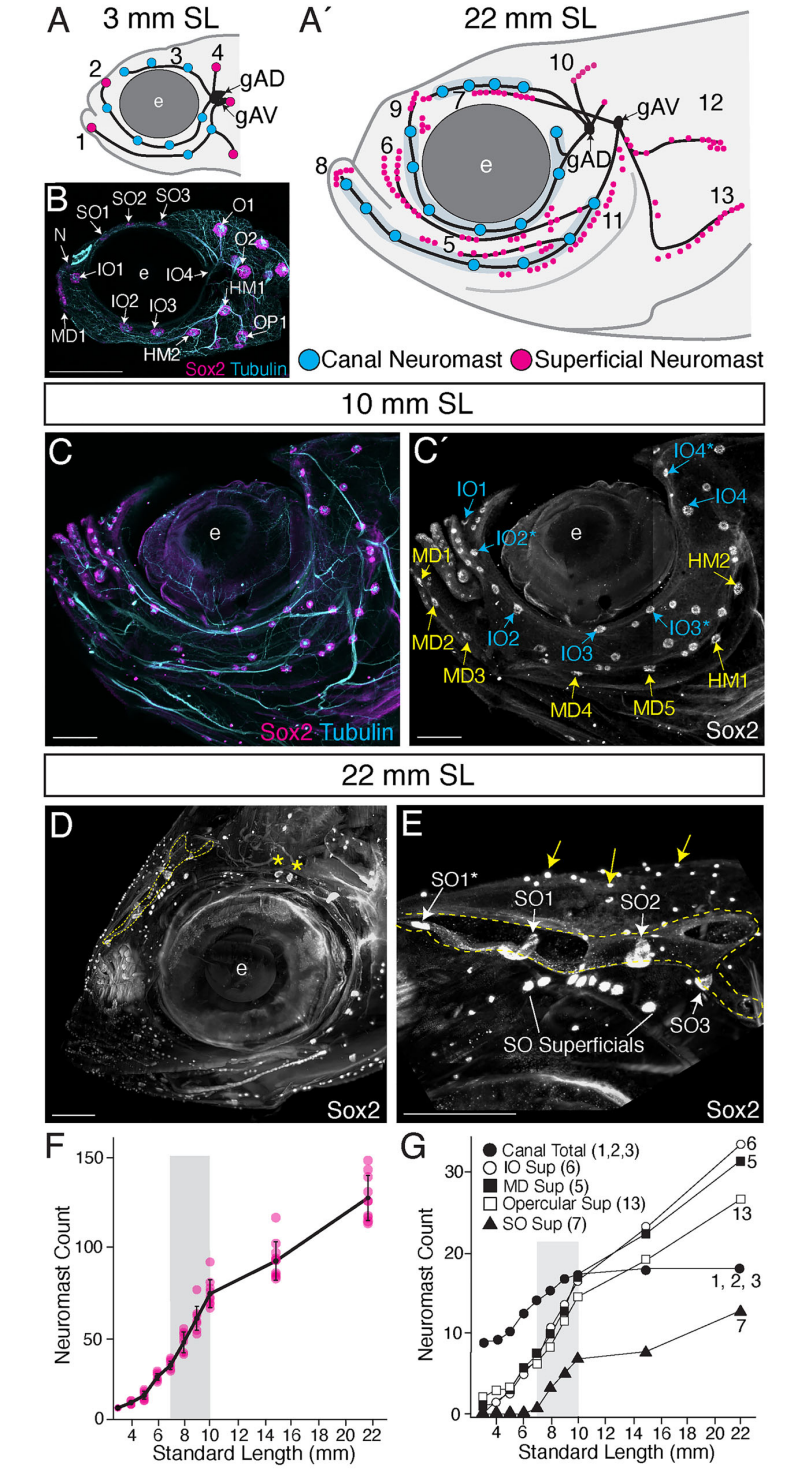
Development of neuromast patterning in the zebrafish anterior lateral line system from early larval to adult stages (3–22 mm SL). (A–A′) Summary schematic of patterning changes between 3 mm SL/7 dpf larvae and 22 mm SL adults. The number and size of canal neuromasts (blue) increase, and hundreds of superficial neuromasts (magenta) are added. Each canal line and superficial neuromast series found in the adult is labeled 1–13. (A) At 3 mm SL, the presumptive mandibular (MD) canal line (1), infraorbital (IO) canal line (2), supraorbital (SO) canal line (3), and otic series (4) are present. (A′) At 22 mm SL, nine additional lines of superficial neuromasts are present: the MD superficial line (5), which runs parallel to the MD canal line; the IO superficial line (6), which runs parallel to the IO canal; the SO superficial line (7), which runs parallel to the SO canal line; the MD series (8) at the anterior end of the MD canal; the nasal series (9) at the anterior end of the IO canal; the anterior pit line (10), which buds dorsally from O1; the hyomandibular superficial line (11); the dorsal opercular line (12); and the opercular line (13).  (B–E) Anterior LL neuromast pattern through ontogeny; e, eye. (B) Confocal maximum projections of the cranial region of a 3 mm SL specimen immunolabeled with Sox2 (magenta) and the nerve marker acetylated alpha‐tubulin (cyan), Scale = 200 µm. (C) Confocal maximum projections of the cranial region of a 10 mm SL specimen immunolabeled with Sox2 and acetylated alpha‐tubulin. (C′) Single channel split of the specimen in (C) showing the Sox2 label. Infraorbital (IO) canal neuromasts are labeled in cyan, and hyomandibular/mandibular (HM/MD) canal neuromasts are labeled in yellow. Scale = 200 µm. (D) Lightsheet maximum projections of the cranial region of a 22 mm SL young adult specimen labeled with Sox2. The image was processed with IMARIS. The SO canal boundaries are highlighted (yellow dotted line). Yellow asterisks indicate an example of “splitting” of a canal neuromast. Scale = 100 µm. (E) Close‐up of the supraorbital (SO) canal of the same specimen. The canal boundaries are highlighted (yellow dotted line), and canal neuromasts SO1*‐SO3 are indicated with white arrows and labeled. The SO superficial neuromasts are also indicated. Examples of non‐neuromast Sox2 expression are indicated by yellow arrows. Scale = 100 µm. (F–G) Quantification of neuromast patterning over ontogeny (3–22 mm SL). The region showing neuromast counts from 7–0 mm SL specimens is emphasized (gray rectangle). (F) Neuromast count versus Standard Length (mm) for (*n* = 10 each) of 3–10, 15, and 22 mm SL specimens. Values present in brackets denote standard deviation. (G) Neuromast count by line (total canal, IO superficial, MD superficial, opercular superficial, and SO superficial) versus Standard length (mm).

Much of our knowledge of LL development comes from the zebrafish (*Danio rerio*), where the early development of the posterior LL has been thoroughly characterized at a molecular and cellular level during embryogenesis (the first 3 days post‐fertilization; dpf) (reviewed by Aman and Piotrowski 2011; Dalle Nogare and Chitnis 2017; Piotrowski and Baker 2014). Recently, the systems‐level development of the more complex zebrafish anterior LL has also been described up to 10 dpf (Iwasaki et al. 2020). This work was built on a seminal study on the innervation of the anterior (pre‐otic) and posterior (post‐otic) components of the zebrafish LL system (Raible and Kruse 2000). Raible and Kruse (2000) established that the LL nerves have stereotypical entry points into the hindbrain, where the anterior LL enters with the facial (seventh) motor nerve root, while the posterior LL enters with the glossopharyngeal (ninth) nerve. Moreover, they found that the zebrafish anterior LL system comprises separate anterodorsal (AD) and anteroventral (AV) systems, which they inferred originate from AD anterodorsal and anteroventral placodes (located anterior to the otic vesicle), as postulated by Northcutt (1992). Each placode gives rise to a migrating primordium of neuromast progenitor cells (which later deposit neuromasts at stereotyped locations) as well as the ganglion that innervates these cells. The early larval AD system consists of the supraorbital (SO) and infraorbital (IO) neuromast lines, above and below the eye, respectively, innervated by the anterodorsal ganglion (gAD). The AV system consists of the continuous preopercular and mandibular (MD) lines along the jaw, and the anteroventral ganglion (gAV). The overall organization of the anterior LL is schematized in Figure 1A,A′.

Raible and Kruse's (2000) study focused specifically on the embryonic and early larval stages of zebrafish development, up to 5 dpf. Following the embryonic establishment of LLs from placode‐derived primordia, we know that additional neuromasts are added to the posterior LL through one of three different mechanisms. The first mechanism relies on migration of new primordia: the posterior LL forms secondary primordia (prim II and primD) at 48 hpf that migrate parallel to the original line ventrally and dorsally, respectively, dropping off superficial neuromasts in their wake (Sarrazin et al. 2010). The second mechanism, intercalation, occurs in larval stages beginning at 3–4 dpf. In this mechanism, interneuromast progenitors, which are deposited between existing neuromasts during the initial migration of the primordia, proliferate and condense into new neuromasts (Ledent 2002). The third mechanism, budding, occurs at juvenile stages. In this mechanism, cells from a founder neuromast proliferate, extend, and then round up to condense into a daughter neuromast (Ledent 2002; Wada et al. 2013).

Similar mechanisms were described by Iwasaki et al. (2020) in the first 10 days of anterior LL development. However, the entire LL system expands enormously during subsequent ontogeny. As the animal continues to grow from larval stages to adulthood, early forming presumptive canal neuromasts increase in size and become enveloped in grooves that later ossify into canals (Webb and Shirey 2003). Simultaneously, hundreds of smaller superficial neuromasts emerge over the entire fish, including in the cranial region, to form complex patterns on the surface of the skin (Ghysen and Dambly‐Chaudière 2004; 2007; Webb and Shirey 2003). The developmental mechanisms underlying the addition of these superficial neuromasts remain unknown. However, for the posterior LL system it has been shown that innervation is dispensable during embryonic development but becomes necessary by juvenile stages for the budding process that produces vertical “stitches” of superficial neuromasts: when the posterior LL ganglion is unilaterally ablated from juvenile zebrafish, the budding process fails to occur, such that no superficial neuromasts develop along the flank on the experimental side (Wada et al. 2013). In the anterior LL, only the superficial opercular line has had neuromast ontogeny described in detail (Wada et al. 2010), and the ontogeny of complete adult anterior LL innervation has not been described for any teleost to date.

Here, by combining live imaging of transgenic markers with CUBIC clearing and immunolabeling methods, we visualized zebrafish anterior LL development from early larval to adult stages at high spatiotemporal resolution. We find that late‐forming superficial anterior LLs form parallel to lines of presumptive canal neuromasts during early larval development, with each superficial line innervated by a new branch of the AV ganglion. Many of the newly forming superficial neuromasts develop through previously documented strategies: primordium migration, budding, and intercalation. However, in the case of the SO superficial line, we describe a novel, hybrid‐origin mechanism of superficial neuromast line formation that combines neuromast tissue from the AD system with innervation from the AV system. Using laser ablation of the ganglia, we establish that functional removal of anterior LL innervation decreases superficial neuromast number and disrupts patterning. Specifically, canal neuromast growth is limited, and a subset of superficial neuromasts, including those formed by the newly described hybrid‐origin mechanism, are absent. Together, our findings reveal that a “developmental switch” occurs at late larval stages, after which innervation is broadly required for anterior LL development.

## 2 Materials and Methods

### 2.1 Animal Husbandry

Zebrafish (*D. rerio*) were maintained in accordance with IACUC‐approved protocols at the University of Chicago. Embryos and early‐stage larvae were maintained in E3 solution (5 mM NaCl, 0.17 mM KCl, 0.33 mM Ca_2_Cl_2_, 0.33 mM MgSO_4_), which was supplemented with 0.3% 1‐phenyl‐2‐thiourea (PTU; Sigma) to block pigment formation for analyses between 24 hpf and 7 dpf, and staged according to standard guidelines (Kimmel et al. 1995). Later stage larvae (up to 10 mm standard length [SL]), juveniles (11–22 mm SL), and adults (> 3 months and >22 mm SL) were staged by measuring SL. SL was measured from the tip of the snout to the caudal peduncle (Parichy et al. 2009), providing a more accurate measure of developmental stage than dpf, because growth rates differ between individuals (see Table 1 for stages analyzed in this study). Embryos were obtained from crosses of adult fish stocks of wild type (*AB line) and/or transgenic lines. The following transgenic lines were used: *Tg(CldnB:GFP)^zf106^* is a tight junction marker that labels cell membranes in the LL system, other placodes, and epithelia (Haas and Gilmour 2006); *Tg(Hgn39d:GFP)^nkhgn39dET^* is an enhancer trap insertion (Nagayoshi et al. 2008) into the *contactin‐associated protein 2a* gene, which specifically labels LL afferent nerves as well as the lens (Faucherre et al. 2009; Pujol‐Martí et al. 2012).

**TABLE 1:** Zebrafish stages of ontogeny analyzed in this study. Based on Parichy et al. (2009) and our observations.

Standard length (SL)	Approx. time in weeks post‐fertilization (wpf)	Life stage
3 mm	∼1	Larval
4 mm	∼2	
5 mm	∼3	
6 mm	∼3‐4	
7 mm	∼4‐5	Late larval
8 mm	∼4‐5	
9 mm	∼5	
10 mm	∼5–6	
11 mm	∼5–7	Juvenile
12 mm	∼6–7	
15 mm	∼6–8	
22 mm	∼11–12	
> 22 mm	> 12	Adult

### 2.2 Vital Dye Labeling and Live Imaging

The vital dye Tetramethylrhodamine Ethyl Ester, Perchlorate (TMRE; Invitrogen) labels the mitochondria‐rich hair cells to reveal the neuromasts. Zebrafish were incubated in 5 nM TMRE in E3 medium: larvae were incubated for 30 min and adults for up to 1 h (Esterberg et al. 2013; Mandal et al. 2018). After incubation, the specimens were washed repeatedly in E3 medium to remove excess TMRE. Specimens were anesthetized using 0.08%–0.1% tricaine in E3 for a maximum of one hour after TMRE treatment to allow imaging. Fish were monitored continuously and immediately removed from the tricaine solution if opercular movements, indicative of respiration, stopped. TMRE‐labeled specimens were live imaged using a Leica M205A epifluorescent microscope with an AmScope MU500‐PB10 5MP USB Camera or a Zeiss LSM 900 confocal with the Plan‐Apochromat 10×/0.3 W or 40×/1 W objectives. For epifluorescent imaging, specimens were positioned in a 35 mm petri dish of E3 medium containing a lateral or dorsal zebrafish larval body shape agarose mold. Custom 3D printed mold blocks were designed in Tinkercad and printed with the Ultimaker 3 printer in PVA material (gift from Elaine Kushkowski). Molds were made by placing the mold blocks in 1%–1.5% agarose for 30 min before carefully removing them with forceps. Confocal imaging was performed on larvae embedded in 0.7%–1% agarose (Invitrogen UltraPure Low Melt Agarose Cat #16500) dissolved in E3 medium containing 0.08%–0.1% tricaine, cooled to 37°C in 60 mm glass bottom dishes (MatTek, USA). Larvae mounted in agarose were carefully positioned and removed from the agarose after imaging using a wire tool. Larvae that were previously stained with TMRE were then housed in separate tanks. Images were acquired using the Zeiss Zen software, and image post‐processing was performed in FIJI (Schindelin et al. 2012).

### 2.3 CUBIC Clearing and Immunolabeling

For adult zebrafish immunolabeling, a CUBIC clearing and staining protocol was adapted from (Pende et al. 2020) on specimens fixed in 4% paraformaldehyde (PFA; Sigma). Adult specimens were depigmented in cold acetone overnight at ‐20°C before lightly bleaching for 5–20 min in a 3% solution of hydrogen peroxide in 1% KOH in water. Specimens were incubated overnight in Low Urea CUBIC I (Pende et al. 2020) solution at 37°C for initial clearing. For immunolabeling, cleared specimens were blocked using goat serum at 25°C for 3–4 h before incubation with the primary or secondary antibody. Both primary and secondary antibody solutions were centrifuged for 5 min to remove aggregates immediately before incubation. Incubation times with primary antibody varied from 1 to 4 days at 37°C, depending on specimen size, followed by incubation with secondary antibody from 1–3 days at 37°C. Specific incubation times based on specimen size were as follows: 4–6 mm SL: 1 day primary, 1 day secondary; 6–10 mm SL: 2 days primary, 1–2 days secondary; 10–22 mm SL: 3 days primary, 2–3 days secondary. To preserve transgenic fluorescence of *Tg(Hgn39d:GFP)* specimens, bleaching was limited to < 10 min. Cleared and stained specimens were deskinned with forceps to remove unwanted signal from epithelial tissue before imaging of the underlying neuromasts and nerves. Specimens were placed in CUBIC R + (N) RI matching solution (Kubota et al. 2017) for at least 30 min before imaging and mounted between two glass coverslips. Cleared and stained specimens were imaged on either a Zeiss LSM 900 microscope or a LaVision Large Format Lightsheet microscope.

For embryonic and early larval stages, immunolabeling was performed as previously described (Prince et al. 1998) on specimens fixed in 4% PFA. The primary antibodies used were anti‐Sox2 1:200 (anti‐rabbit; Genetex GTX124477; RRID AB_1117806), anti‐GFP 1:200 (anti‐rabbit; Thermo Fisher Cat # A‐6455; RRID: AB_221570), and anti‐acetylated alpha‐tubulin 1:200 (anti‐mouse; Sigma‐Aldrich Cat# T7451; RRID_AB_609894). Secondary antibodies used were Alexa 488, 1:500 (Invitrogen anti‐mouse A‐11001; anti‐rabbit A‐11008), Alexa 546 1:500, (Invitrogen anti‐mouse A‐11003; anti‐rabbit A‐11035), and Alexa 633, 1:300 (Invitrogen anti‐mouse A‐21052; anti‐rabbit A‐21070). Specimens were mounted in 1% agarose in 60 mm dishes and imaged on the Zeiss LSM 900 confocal with the Plan‐Apochromat 10×/0.3 W or 40×/1 W objectives.

### 2.4 Combined Use of Transgenic Lines and Immunolabeling

We made extensive use of *Tg(CldnB:GFP)* and *Tg(Hgn39d:GFP)* specimens, either live or fixed, throughout this study. We also used *Tg(CldnB:GFP;Hgn39d:GFP)* double transgenic specimens; however, a caveat of this approach is that both LL progenitors and innervation are labeled with the same fluorophore, making it challenging to discriminate LL cells from either epithelial cells or the nerves at late stages. To rectify this issue, we immunolabeled fixed specimens with anti‐Sox2 antibody (Genetex GTX124477, RRID AB_1117806), a label of LL primordia and interneuromast cells (Hernández et al. 2007). While Sox2 is a transcription factor, and the antibody therefore labels nuclei of progenitors as expected, we find that this antibody additionally labels emerging LL nerves (confirmed by co‐labeling with acetylated alpha‐tubulin). Sox2 labeling thus clarifies whether cells visualized with the double transgene marker are LL cells, nerves, or neither.

### 2.5 Terminology Used to Describe LL Nerves

We have modeled the terminology we use to describe the anterior LL nerves, including those that project to superficial lines of neuromasts, closely on the format used by Raible and Kruse (2000). Our terms are defined as: **nADso**, nerve from the **AD** ganglion projecting to the **s**upra**o**rbital canal line of neuromasts; **nAVsos**, nerve from the **AV** ganglion projecting to the **s**upra**o**rbital **s**uperficial line of neuromasts; **nAVmd**, nerve from the **AV** ganglion projecting to the **m**an**d**ibular canal line of neuromasts; **nAVmds**, nerve from the **AV** ganglion projecting to the **m**an**d**ibular line of **s**uperficial neuromasts; **nADio**, nerve from the **AD** ganglion projecting to the **i**nfra**o**rbital canal line of neuromasts; **nAVios**, nerve from the **AV** ganglion projecting to the **i**nfra**o**rbital **s**uperficial line of neuromasts; **nAVop**, nerve from the **AV** ganglion projecting to the **op**ercular line of neuromasts; **nAVopd** nerve from the **AV** ganglion projecting to the **op**ercular line of neuromasts, **d**orsal branch; **nADap** nerve from the **AD** ganglion projecting to the **a**nterior **p**it line of neuromasts; **nAVhm** nerve from the **AV** ganglion projecting to the **h**yo**m**andibular line of neuromasts.

We note that the nADio line was previously referred to by Raible and Kruse (2000) as nADb (with “b” indicating buccal ramus), but we have instead elected to use terminology that emphasizes the relationship of this nerve with the IO neuromasts. Similarly, the nAVios line was previously referred to by Iwasaki et al. (2020) as nAVmx (with “mx” indicating maxillary), but we have again elected to use terminology that emphasizes the relationship of this nerve with the IO superficial neuromasts.

### 2.6 Large Format Lightsheet Imaging

Juvenile and adult specimens were imaged on a LaVision Large Format Lightsheet, using a 4× objective, and 2.0 µM excitation sheet in CUBIC R+ (N) imaging medium (Kubota et al. 2017).

### 2.7 Laser Ablations

A laser‐scanning confocal microscope (either Zeiss LSM 900 or LSM 710) at 40x magnification was used to unilaterally ablate the anterior LL ganglion (both dorsal and ventral components) of *Tg(Hgn39d:GFP)* specimens. *Tg(Hgn39d:GFP)* specimens were selected for ablation at 4 dpf based on a bright GFP signal in the ganglia coupled with strong swimming ability, indicative of healthy and robust specimens. Larvae were anesthetized and mounted as described above. The side to be ablated was selected at random. A 405 nm laser was used on 60%–75% power to ablate 2–4 cells at once (Morsch et al. 2017). Cells were identified, and the zoom feature in Zen was used to magnify them by 20× (800× total magnification) before exposing them to the laser in six bursts of 30 s, using the photobleaching module in Zeiss Zen software. Animals that received a sham surgery of intense UV laser light applied immediately adjacent to the anterior LL ganglia showed no change in neuromast or innervation patterns at the 6‐week post fertilization (wpf) endpoint (*n* = 5). In successful ablations, all signal from the ganglion cells was permanently removed, and degradation of the LL nerves could be visualized immediately. Larvae in which the anterior LL ganglia had been ablated were raised in groups of ≤ 6 in small tanks of ∼200 mL water volume with daily water changes of 5%–10%. The fish were stained with TMRE each week as described above until sacrifice for final analysis at 6 weeks of age or a minimum length of 11 mm SL. During analysis, superficial neuromasts in the nasal line were not included in the total counts, due to consistent ectopic reinnervation of ablated side nasal neuromasts by control side nerves.

### 2.8 Image Processing

LL tissue was segmented out from epithelia in selected confocal image stacks (used in Figures 4 and 5) of transgenic *Tg(CldnB:GFP)* or *Tg(CldnB:GFP;Hgn39d:GFP)* larval specimens using the software FIJI. Image stacks were thresholded and converted to binary. The “analyze particles” tool was used to generate masks of image features 50 µm^2^ or larger. Masks were manually edited to exclude LL tissue using the “watershed” and “flood fill” tools and enlarged using the “dilate” tool. To remove the epithelial tissue signal, masks were subtracted from the original image stack using the image calculator tool. To compare signal intensity between control and experimental specimens in Figures 6 and 7, images were taken at the same laser power and processed using identical parameters. In specimens immunolabeled with Sox2, canal neuromast length was measured transverse to the canal, and width was measured parallel to it, as described by Webb and Shirey (2003), to establish aspect ratios.

## 3 Results

### 3.1 The Zebrafish Anterior LL System Expands by the Addition of Lines of Superficial Neuromasts

The ontogeny of the zebrafish anterior LL system at late larval and juvenile stages has not been described in detail. This gap in knowledge reflects the challenge of visualizing structures such as cranial nerves in juvenile zebrafish that become increasingly opaque through development. In this study, we have visualized zebrafish anterior LL development in larval, juvenile, and young adult specimens at high temporal and spatial resolution by combining live imaging of transgenic markers with CUBIC clearing and immunolabeling methods. To map out the postembryonic development of the anterior LL, we documented changing neuromast patterns and their innervation in larvae of 3 mm SL (7 dpf) through juvenile stages and on to adults of 22 mm SL (approximately 3 months post‐fertilization; mpf), as shown in Figure 1.

As schematized in Figure 1A, in 3 mm SL larvae there are just four lines of neuromasts present: the presumptive mandibular (MD) canal line (including the hyomandibular (HM) neuromasts) (1), the presumptive infraorbital (IO) canal line (2), the presumptive supraorbital (SO) canal line (3), and the superficial otic series (O) (4). In 22 mm SL adults, the canals have formed, and an additional nine superficial lines or series of neuromasts are present (Figure 1A′, lines 5–13; Table 2). The superficial lines include three that parallel the original canal lines (the MD (5), IO (6), and SO (7) superficial lines), as well as neuromast series that form at the anterior ends of the IO and MD canals via neuromast budding (8–9) (Table 2).

**TABLE 2:** Larval ontogeny of canal and superficial neuromast development.

Canal neuromast	Stage of formation	Mechanism of formation	Where described
SO1*	Larval; 5 mm SL	Buds from SO1	Webb and Shirey (2003) Mechanism described in this study
SO1	Embryonic; 2 dpf	Buds anteriorly from SO2	Iwasaki et al. (2020)
SO2	Embryonic; 1.5 dpf	Forms from migrating SO primordium	Iwasaki et al. (2020)
SO3	Larval; 4 dpf	Intercalates behind SO2	Iwasaki et al. (2020)
			
IO1	Embryonic; 3 dpf	Intercalates between IO2 and superficial neuromast N	Iwasaki et al. (2020)
IO2*	Larval; 6 mm SL	Buds anteriorly from IO2	This study
IO2	Embryonic; 1.5 dpf	Forms from migrating IO primordium	Iwasaki et al. (2020)
IO3	Larval; 4 dpf	Intercalates between IO3 and O2	Iwasaki et al. (2020)
IO3*	Larval; 10 mm SL	Intercalates between IO3 and IO4	This study
IO4	Embryonic; 3 dpf	Forms from migrating IO primordium	Iwasaki et al. (2020)
IO4*	Larval; 8 mm SL	Buds dorsally from IO4	This study
			
HM1	Embryonic; 3 dpf	Forms in place from HM primordium	Iwasaki et al. (2020)
HM2	Embryonic; 3 dpf	Forms in place from HM primordium	Iwasaki et al. (2020)
MD5	Larval; 9 mm SL	Intercalates along MD line	This Study
MD4	Larval; 8 mm SL	Intercalates along MD line	This study
MD3	Larval; 6 mm SL	Intercalates along MD line	Webb and Shirey (2003) Mechanism described in this study
MD2	Larval; 5 mm SL	Intercalates along MD line	Webb and Shirey (2003) Mechanism described in this study
MD1	Larval; 7 mm SL	Intercalates along MD line	Webb and Shirey (2003) Mechanism described in this study
Superficial neuromast line/series	Stage of formation	Mechanism of formation	Where described
IO superficial line	Primordium by 3 dpf; first neuromast 4 mm SL/∼10 dpf	Primordium splits from HM prim	Iwasaki et al. (2020); termed “maxillary” line Larval development is described in this study
MD superficial line	Primordium by 4 dpf; first neuromast 4 mm SL/∼11 dpf	Primordium splits from HM prim	This study
SO superficial line	First neuromast 7 mm SL	Tissue from IO4 and O2; See Figure 5	This study
Otic series	Embryonic; 1.5 dpf	O1 and O2 designated as “homegrown”—develop close to AD primordium	Iwasaki et al. (2020)
Opercular Line	First neuromast by 3 dpf	Budding along subopercular bone growth axis	Full ontogeny of the main branch described in Wada et al. (2010); dorsal branch described by Iwasaki et al. (2020)
Nasal Line	First neuromast by 3 dpf	Medial budding from superficial neuromast N	N described by Iwasaki et al. (2020) Mechanism described in this study
Mandibular series	First neuromast by 3 dpf	Medial budding from superficial neuromast MD1	Series noted in Webb and Shirey (2003) Mechanism described in this study
Anterior Pit Line	First neuromast by 8 mm SL	Buds dorsally from O1	This study Homologous to the anterior pit line seen in other ray‐finned fishes (Actinopterygii), including the teleost channel catfish (Northcutt et al. 2000)

The data that underlie these schematics are shown in Figure 1B–E. At the larval starting point (3 mm SL; Figure 1B), there are stereotypically fourteen neuromasts, connected by simple LL nerves. By late larval stages (10 mm SL; Figure 1C, C′), the system has expanded dramatically to include approximately 100 neuromasts and increasingly branched cranial nerves. At this stage, developing presumptive canal neuromasts are easily distinguished from superficial neuromasts by their larger size (Brown et al. 2023; Webb and Shirey 2003). In Figure 1C′, the individual presumptive canal neuromasts of the IO system are labeled in blue, and those of the preopercular/MD canal (hereafter referred to as the MD canal) are labeled in yellow.

By early adulthood (22 mm SL), the number of superficial neuromasts has increased further, and the canal neuromasts are becoming enclosed by ossifying canals (Webb and Shirey 2003; Figure 1D,E; yellow dotted outlines indicate the canal boundaries). In Figure 1E, the canal neuromasts of the SO system, SO1*‐SO3, are indicated (white arrows; note that SO1* buds from existing neuromast SO1). Typically, the canal neuromasts lie at the bottom of the bony canals, curving upward along the sides of the canal, and elongating perpendicular to the canal as the fish grows (Webb and Shirey 2003). By contrast, superficial neuromasts are significantly smaller and lie adjacent to the canals on the surface of the skin (SO superficial neuromasts are indicated, Figure 1E). There are also much smaller clusters of cells labeled by Sox2, which we assign as taste buds (Figure 1E, yellow arrows; Germanà et al. 2009; Hansen et al. 2002).

We quantified the total anterior LL neuromast count on one side of the head between 3 and 22 mm SL stages, using the vital dye hair cell label TMRE to label the neuromasts repeatedly as ontogeny proceeded (Figure 1F; *n* = 10). Throughout ontogeny, there is a consistent increase in total neuromast number with body size, with variation in neuromast number between specimens increasing over time. Notably, there is a sharp increase in neuromast addition rates at late larval stages (7–10 mm SL) (Figure 1F; gray panel). Canal neuromasts and superficial neuromasts, making up superficial neuromast lines, are added with different kinetics, as shown in Figure 1G. About half of the presumptive adult canal neuromasts are added at stages between 4 and 9 mm SL, with the last forming canal neuromasts developing just before the onset of formation of the bony canals beginning at 10–11 mm SL (Webb and Shirey 2003). Across the superficial lines, the broad coordination in neuromast addition likely reflects consistent growth of the underlying dermal structures. The spike in superficial neuromast addition rates occurs across all major superficial lines at 7–10 mm SL (Figure 1G; gray panel). Notably, this is also when the peak phase of metamorphic remodeling at the larva‐to‐juvenile transition occurs (McMenamin and Parichy 2013; Webb and Shirey 2003).

Our neuromast quantifications allowed us to track the ontogeny of each canal neuromast and neuromast line; these data are summarized in Table 2. As noted by Webb and Shirey (2003), not all neuromasts that form at embryonic stages are destined to become canal neuromasts, as exemplified by the early‐forming superficial otic series and neuromast MD1, which remains within the epidermis into adulthood. In the adult zebrafish, anterior LL canal neuromasts were described by Webb and Shirey (2003) as being organized into four neuromasts of the SO canal, five neuromasts of the IO canal, and five neuromasts of the MD canal, with pores in the canal bones lying in between adjacent neuromast organs and thus connecting the canal with the external environment. By contrast, we found about seven neuromasts of the IO canal and about seven neuromasts of the MD canal. This discrepancy likely reflects, at least in part, our finding that neuromasts with canal‐like morphology are present at the termini of each canal, which remain unroofed, in addition to lying between the pores as previously described. It is also possible that different zebrafish lab strains might exhibit differences in canal neuromast number. Of relevance, we find some variation in canal neuromast number between individuals, and in adults, we have found that canal neuromasts sometimes split into two adjacent structures (e.g., Figure 1D, asterisks).

**FIGURE 2:**
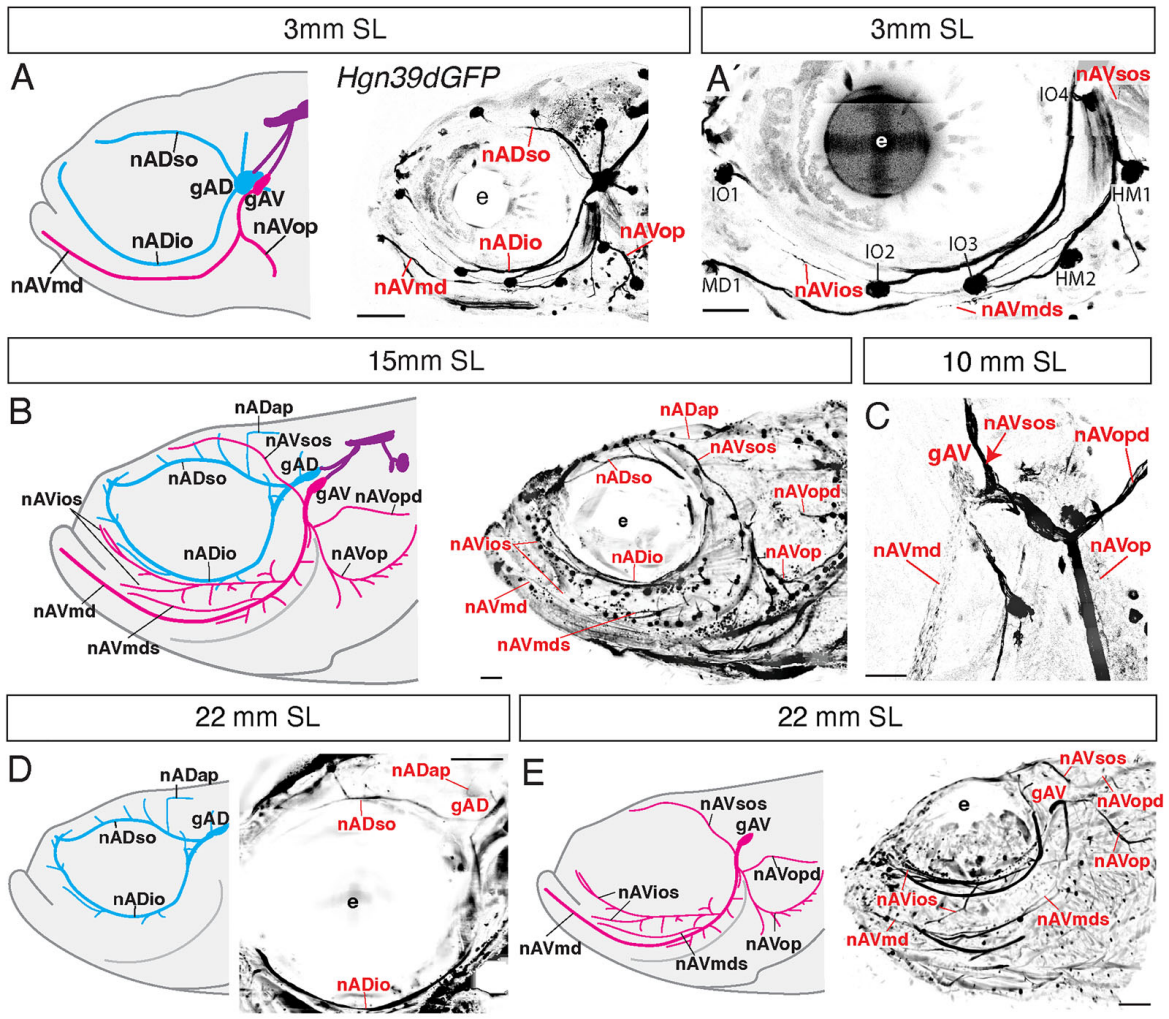
Description and development of adult zebrafish anterior lateral line innervation. (A–A′) Innervation map at early larval stages (3 mm SL/7 dpf). (A) 3 mm SL nerve map schematic, based on adjacent image of anterior lateral line afferent innervation of a 3 mm SL specimen immunolabeled for anti‐GFP against *Tg*(*Hgn39d:GFP*) (confocal maximum projection). The schematic shows the anterodorsal nerves (nAD) and ganglia in cyan (nAD supraorbital [so] and infraorbital [io]) and the anteroventral nerves (nAV) in magenta (nAV mandibular [md] and opercular [op]). Axonal projections to the hindbrain are indicated in purple. (A′) Close up of late forming superficial nerves (red labels) in the infraorbital region, running parallel to canal nerves, and neuromasts (black labels) at 3 mm SL. (B) 15 mm SL nerve map schematic showing the mature anterodorsal system (cyan: nAD supraorbital, infraorbital, and anterior pit [ap]) and anteroventral system (magenta: nAV mandibular, mandibular superficial [mds], infraorbital superficial [ios], opercular, opercular dorsal [opd], and supraorbital superficial [sos]), based on adjacent image of innervation of a 15 mm SL specimen immunolabeled for anti‐GFP against *Tg*(*Hgn39d:GFP*) (lightsheet maximum projection). (C) 10 mm SL specimen, showing nerve branch points from the anteroventral ganglion (confocal maximum projection at high magnification). The nAVsos branch point is indicated with a red arrow. Scale = 50 µm. (D–E) Innervation map at adult stages (22 mm SL) immunolabeled for anti‐GFP against *Tg*(*Hgn39d:GFP*). (D) Lightsheet maximum projection of orbital region of the cranium showing the anterodorsal ganglion and nerves (cyan in adjacent schematic). (E) Lightsheet maximum projection of lateral‐ventral view of a 22 mm SL specimen showing the anteroventral nerves (magenta in adjacent schematic). These adult specimens were cleared with CUBIC and immunolabeled with anti‐GFP antibody to enhance the endogenous *Tg(Hgn39d:GFP)* signal. Scale = 100 µm.

We next described the second essential component of the anterior LL system, its sensory innervation from the gAD and gAV. Figure 2 shows confocal and lightsheet maximum projections of cleared and stained specimens that reveal the ontogeny and adult pattern of anterior LL innervation in *Tg(Hgn39d:GFP)* specimens. *Tg(Hgn39d:GFP)* is a transgenic line that selectively labels LL afferent nerves (Faucherre et al. 2009; Pujol‐Martí et al. 2012), with individual neuromasts recognizable by the complex dendritic arbors that contact the hair cells (Faucherre et al. 2009). Anterior LL innervation is divided into the AD system, innervated by gAD, and the AV system, innervated by gAV.

In 3 mm SL specimens, the AD innervation is relatively simple, as schematized in Figure 2A and shown by the adjacent Hgn39dGFP nerve map. The embryonic AD system (cyan in Figure 2A) consists of branches innervating the SO and IO presumptive canal lines. The embryonic AV system (magenta in Figure 2A) innervates the MD and Op (opercular) lines. Both ganglia project axons into the central nervous system at the level of the hindbrain (purple) (Figure 2A). Figure 2A′ shows a higher magnification view of the 3 mm SL specimen, revealing the first developing nerve fibers for late‐forming superficial lines that will branch from the gAV (red labels). Note that in the descriptions that follow, we build on terminology established by Raible and Kruse (2000), adding the designation “s” for the newly forming nerves that will innervate superficial lines of neuromasts (see Section 2.5 for details of terminology). In the 3 mm SL specimen, the nAVios branches from near neuromast HM1 and runs parallel to the IO nerve; the nAVmds branches anteriorly from neuromast HM2 and runs part of the way down the MD nerve; and the nAVsos branches dorsally from the gAV root above neuromast HM1. We use the nomenclature of HM1 and HM2, based on Iwasaki et al. (2020), to describe the embryonic neuromasts that will eventually become canal neuromasts in the preopercular portion of the MD canal.

By juvenile stages (15 mm SL), the fish has entered a post‐metamorphic growth phase, and all branches of adult innervation have been established (Figure 2B). The organization of the AD system (cyan) remains very similar to that of 3 mm SL specimens (compare schematics in Figure 2A,B). By contrast, the AV system (magenta) is significantly expanded at this later stage. New nerves have been added running parallel to each of the existing canal lines, and a secondary dorsal branch of the opercular line (nAVopd) has formed (Figure 2B). By these juvenile stages, nAVios has split into a dorsal branch, which innervates superficial neuromasts parallel to the IO line, and a ventral branch, which innervates superficial neuromasts parallel to the MD line. Iwasaki et al. (2020) previously noted the early origins (up to 10 dpf) of two of these nerves, nAVopd and nAVios (which they designated as nAVmx; see Section 2.5). Figure 2C shows a close‐up view of the root of the AV system dividing into several branches at late larval stages (10 mm SL), confirming that late‐forming nerves such as the nAVsos (red arrow) originate from the gAV. The AD (Figure 2D) and AV (Figure 2E) systems are separately shown in a 22 mm SL adult, with accompanying schematics highlighting the relative elaboration of each system's innervation. The expansion of the anterior LL occurs primarily due to the addition of AV‐innervated superficial neuromast lines at larval stages.

### 3.2 Neuromasts Are Added by Continued Use of Embryonic Mechanisms at Later Ontogenetic Stages

Next, we investigated the mechanisms that drive the formation of the superficial lines that lie parallel to the presumptive canal lines, as well as the proliferation of presumptive superficial and canal neuromasts at larval stages. Postembryonic descriptions of the zebrafish posterior LL have described three different mechanisms by which late‐forming neuromasts can develop: migration of new primordia (Sarrazin et al. 2010), intercalation (Ledent 2002), and budding (Ledent 2002; Wada et al. 2013). We asked if, when, and how each of these mechanisms might be used in the zebrafish anterior LL system, as well as whether there are any anterior LL‐specific mechanisms of superficial neuromast formation.

**FIGURE 3:**
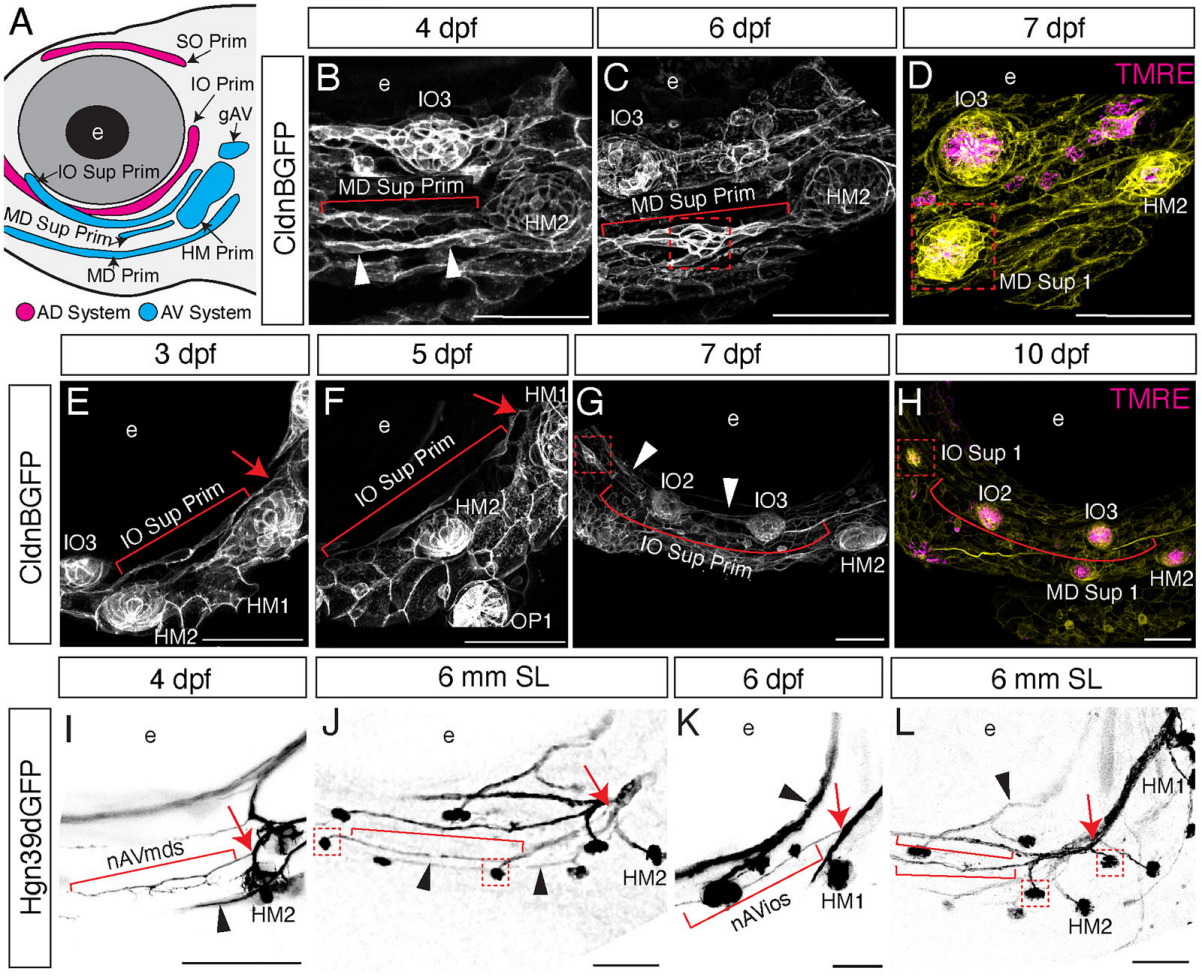
Mechanisms of larval superficial neuromast formation from late‐forming primordia. (A) Schematic of CldnB‐labeled lateral line primordia derived from AD (magenta) and AV (cyan) placodes. (B–H) Confocal images of live *Tg(CldnB:GFP)* specimens. (B–D) Development of the mandibular superficial line from its migrating primordium (MD Sup Prim) at (B) 4 dpf; (C) 6 dpf; and (D) 7 dpf. (B) The mandibular superficial primordium (red bracket) extends from HM2 by 4 dpf, parallel to the mandibular primordium (white arrowheads). (C) By 6 dpf the primordium has begun to condense into a protoneuromast (red box). (D) At 7 dpf, the first neuromast of the MD superficial line is in place (MD Sup 1; red box); *CldnB:GFP* (yellow); TMRE (magenta) labels hair cell mitochondria within the neuromast. (E–H) Development of the infraorbital superficial line from its migrating primordium (IO Sup Prim). Extension of the IO superficial primordium (red bracket) adjacent to HM1 at (E) 3 dpf; (F) 5 dpf; (G) 7 dpf; and (H) 10 dpf; where it condenses into the first neuromast of the IO superficial line (IO Sup 1; red box). (E) The infraorbital superficial primordium originates adjacent to HM1 (red arrow) and extends a few cells ventrally. (F) At 4 dpf, the primordium has extended past HM2, running parallel to the IO canal line. (G) At 7 dpf, the primordium has extended parallel to the IO canal line (white arrowheads) to its terminal location adjacent to the eye between IO1 and IO2 (red box), where the terminus has condensed into a protoneuromast. (H) At 10 dpf, this terminus (red box) has differentiated into the first neuromast in the IO superficial line, labeled with *CldnB:GFP* (yellow) and neuromast hair cell marker TMRE (magenta). (I–H) Confocal images of live (I, K) and fixed (J, H) *Tg(Hgn39D:GFP)* specimens immunolabeled for GFP. (I) The nAVmds nerve (red bracket) is shown at 4 dpf, branching from gAV near HM2 (red arrow), and running parallel to the MD canal nerve (black arrowhead). (J) At 6 mm SL, the nAVmds nerve (red arrowheads) has extended anteriorly to innervate neuromasts in the mandibular superficial line (red boxes), continuing to run parallel to the MD canal nerve (black arrowheads). (K) The nAVios nerve (red bracket) branches off from gAV near HM1 (red arrow) and runs parallel to the IO canal nerve (black arrowhead). (L) At 6 mm SL, the nAVios nerve (red arrow) has formed a ventral branch, with both the original and ventral branches (red brackets) running parallel to the IO canal nerve (black arrowhead) and innervating neuromasts in the infraorbital superficial line (red boxes). e, eye.

Figure 3 documents the emergence of lines of superficial neuromasts from newly emerging primordia. By 3 dpf, the MD, IO, and SO primordia of the presumptive canal lines have migrated to their final locations. Next, lines of superficial neuromasts start to develop from newly emerging superficial primordia, all of which derive from the AV system (schematized in Figure 3A; blue). Figure 3B–H shows confocal maximum projections of LL tissue in living zebrafish specimens, imaged using the *Tg(CldnB:GFP*) transgene (Haas and Gilmour 2006). Figure 3B–D documents the emergence of the MD superficial line, which runs parallel to the presumptive MD canal line, from a newly emerging strand of CldnB‐labeled primordium cells that extends anteriorly from the AV‐derived HM system at relevant stages (*n* = 5 specimens). Figure 3B shows a 4 dpf specimen with a new primordium splitting off the anterior end of the HM primordium (red bracket), parallel to the presumptive MD canal line (white arrowheads). By 6 dpf (Figure 3C), this MD superficial primordium has extended partway along the length of the presumptive MD canal line, and cells are beginning to condense into a protoneuromast (red box). At 7 dpf (Figure 3D), the first MD superficial neuromast (MD Sup 1; red box) is in place.

Figure 3E–H documents the similar emergence of the IO superficial line (*n* = 5). At 3 dpf (Figure 3E), a new strand of CldnB‐labeled primordium cells (red bracket) extends anteriorly from a location adjacent to the AV‐derived HM system (red arrow). This IO superficial primordium was described by Iwasaki et al. (2020) as forming the “maxillary line” and our findings corroborate their previous description: we find that the primordium elongates parallel to the presumptive IO canal line (Figure 3G, white arrowheads) at 5 dpf (Figure 3F), and by 7 dpf (Figure 3G) extends anteriorly (red bracket) to an enlarging endpoint (red box), beyond neuromast IO2, where the tissue condenses into the first new IO superficial neuromast (IO Sup 1, red box; Figure 3H) by 10 dpf.

We also used the *Tg(Hgn39d:GFP)* transgene to visualize innervation during the development of the MD and IO superficial neuromast lines (Figure 3I–L). Figure 3I,J shows the emergence and later development of the MD superficial nerve (nAVmds). Figure 3I shows the nerve (red bracket) branching off from the same location on the nAVhm nerve (red arrow) as its primordium, at 4 dpf. By the 6 mm SL stage (Figure 3J), the nerve (red bracket) extends to innervate multiple MD superficial neuromasts (red boxes). Figure 3K,L shows the similar emergence and later development of the IO superficial nerve (nAVios). Figure 3K shows the new nerve branching from the same location on the nAVhm nerve as the newly forming superficial IO line primordium. By the 6 mm SL stage (Figure 3L), the nerve extends to innervate multiple IO superficial neuromasts (red boxes) and has formed a ventral branch that innervates superficial neuromasts parallel to the MD line. In conclusion, the initial establishment of the MD and IO superficial lines occurs over several days, with the system continuing to elaborate throughout ontogeny.

**FIGURE 4:**
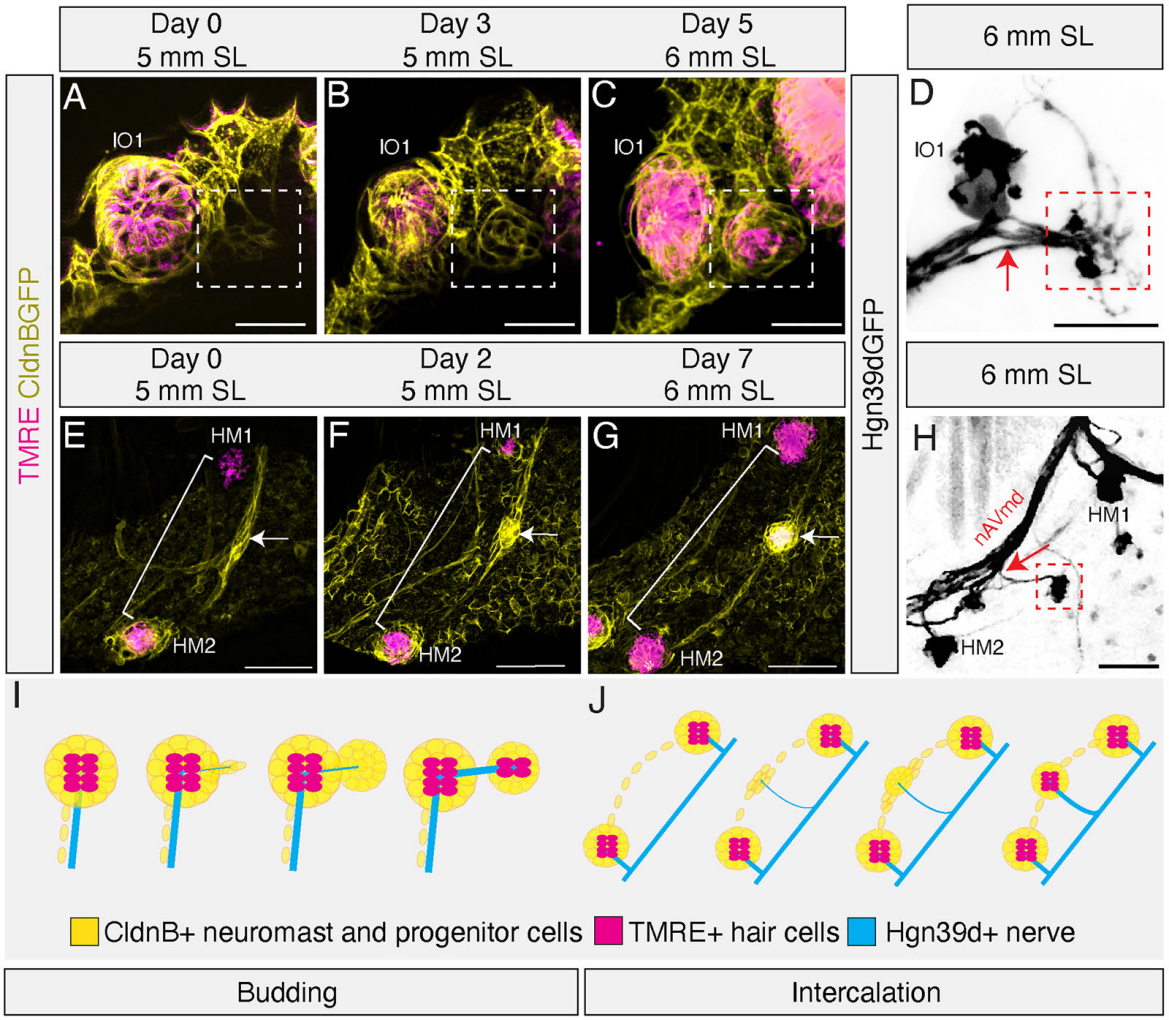
Development of anterior lateral line superficial neuromasts by budding and intercalation mechanisms. (A–C) High magnification confocal time series of lateral line tissue (CldnB:GFP; yellow) and mature neuromasts (TMRE; magenta) showing the budding mechanism of neuromast formation in a single live specimen from 5–6 mm SL. (A) At Day 0, canal neuromast IO1 extends a posterior projection of a few CldnB: GFP‐labeled cells (white box). (B) By Day 3, the cells from the posterior projection have proliferated to form a protoneuromast (white box). (C) By Day 5, this tissue has differentiated into a mature daughter neuromast expressing TMRE (white box) lying directly posterior to the founder neuromast IO1. (D) Confocal image of a live *Tg(Hgn39D:GFP)* 6 mm SL specimen showing a direct nerve branch (red arrow) extending from the ganglion, which also innervates the founder neuromast (IO1), to the daughter (red box) neuromast. (E–G) High magnification confocal time series of the lateral line tissue (CldnB:GFP; yellow) and mature neuromasts (TMRE; magenta) showing the intercalation mechanism of neuromast formation in a single live specimen from 5–6 mm SL. These images were manually segmented to remove unwanted signal from superficial epithelial tissue. (E) At Day 0, a HM superficial neuromast is beginning to form (white arrow) from a thickening of interneuromast tissue (white bracket) between two mature canal neuromasts, HM1 and HM2. (F) At Day 2, the interneuromast tissue has proliferated into a rosette shape (white arrow). (G) By Day 7, the interneuromast tissue has differentiated into a mature superficial neuromast with a TMRE signal (white arrow). (H) Confocal image of a live *Tg(Hgn39D:GFP)* 6 mm SL specimen showing a new nerve branch (red arrow) extending from the main HM nerve between the HM1 and HM2 branches to innervate the newly formed HM superficial neuromast (red box). (I–J) Schematics of neuromast formation processes: CldnB:GFP‐labeled neuromast and progenitor cells (yellow), TMRE+ hair cells (magenta), and Hgn39d+ nerves (cyan). (I) Schematic of the budding process. (J) Schematic of the intercalation process.

Following the establishment of presumptive canal and superficial lines by migrating primordia, many additional neuromasts are added. In Figure 4, we document the stereotyped development of superficial neuromasts via the previously described mechanisms of budding and intercalation, using confocal maximum projections of live specimens (*n* = 5 per mechanism). By repeated co‐labeling of live developing *Tg(CldnB:GFP)* specimens with TMRE, neuromast formation (GFP‐positive; yellow) and subsequent maturation (TMRE‐positive; magenta) were traced over time.

Figure 4A–C tracks an example of the budding mechanism over a 5‐day period. In this 5 mm SL specimen, the IO1 neuromast is just starting to bud at Day 0 (Figure 4A; white box), to produce a new *CldnB:GFP*‐labeled superficial protoneuromast at Day 3 (Figure 4B), with TMRE‐positive (magenta) hair cells developing within the newly formed neuromast by Day 5 (Figure 4C). In Figure 4D, we used *Tg(Hgn39D:GFP)* to document the expansion of innervation associated with the budding process; a direct projection branch (red arrow) from the founder to the daughter neuromast (red box).

Figure 4E–G tracks a HM superficial neuromast (white arrow) in the process of developing between two presumptive canal neuromasts, HM1 and HM2, via intercalation. Figure 4E shows a 5 mm SL specimen in which expansion of *CldnB:GFP* interneuromast progenitors between the presumptive canal neuromasts is just beginning (Day 0; white arrow). By Day 2, a new protoneuromast has formed (Figure 4F; white arrow). By Day 7, TMRE‐expressing hair cells are present in the newly formed superficial neuromast (Figure 4G, white arrow). Importantly, as the fish approaches adulthood, the HM1 and HM2 neuromasts will become enclosed within a bony canal, but the smaller HM superficial neuromasts will remain in the skin. In Figure 4H, we again used *Tg(Hgn39D:GFP)* to document the expansion of innervation associated with the intercalation process; here, a new projection branch (red arrow) from the HM nerve to the newly formed superficial neuromast (red box).

Schematics of neuromast formation by budding and intercalation, respectively, are provided in Figure 4I,J. Taken together, the data we present in Figures 3 and 4 indicate that the increasingly complex patterning of the anterior LL system can be explained, at least in part, by the continued use of embryonic developmental mechanisms at varied locations and developmental stages.

### 3.3 A Novel LL Formation Mechanism With a Hybrid Origin

**FIGURE 5:**
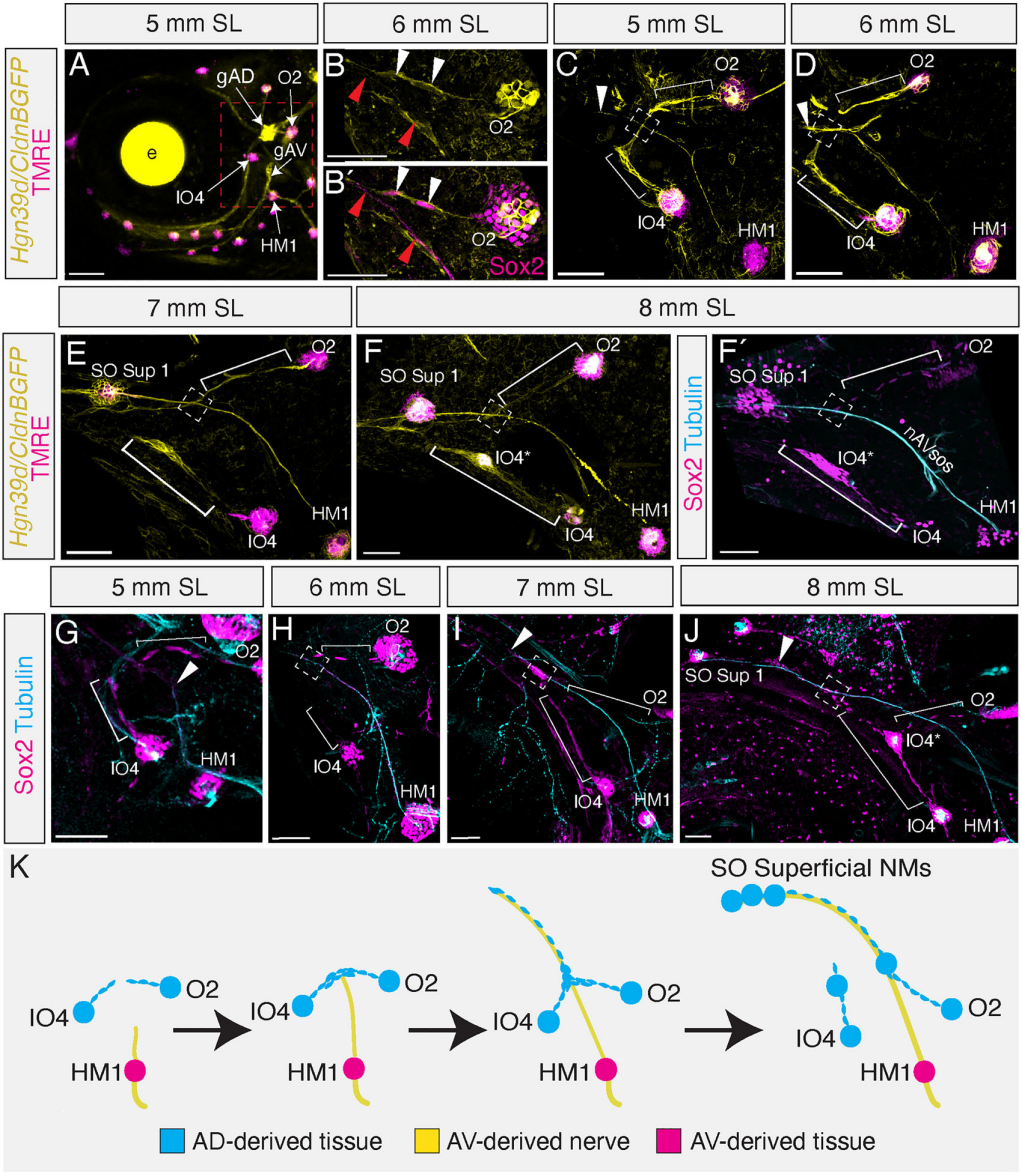
The supraorbital superficial line of neuromast is formed by a novel hybrid‐origin mechanism. (A) Low magnification (10x objective) confocal image of nerves (*Hgn39dGFP*; yellow) and mature neuromasts (TMRE; magenta) at 5 mm SL with the area of interest behind the eye indicated (red box). (B–B′) Comparison of (B) *Tg(CldnB:GFP)* (yellow) and (B′) Sox2 (magenta) expression at the connection between neuromast O2 and the nAVsos nerve (red arrowheads) in a 6 mm SL specimen. Note that the nerve is also Sox2‐positive. CldnB:GFP‐positive cells extending from existing neuromasts are interneuromast cells with Sox2‐positive nuclei (white arrowheads). Scale = 20 µm. (C–F) Live time series images of a single *Tg(CldnB:GFP/Hgn39d:GFP)* (yellow) specimen labeled with TMRE (magenta). In all images, neuromasts HM1, IO4, and O2 are labeled. The intersection between the nerve originating from HM1 (white arrowhead) and strands of interneuromast cell‐derived primordial tissue originating from IO4 and O2 (white brackets) is noted with a white box. Scale = 50 µm. (C) Initial intersection of the nerve and primordia at 5 mm SL. The nAVsos nerve extending from HM1 (white arrowhead) has no lateral line tissue along it and intersects (white box) with the CldnB:GFP‐labeled primordium extensions (yellow) from O2 and IO4 (white brackets). (D) Adherence of primordium tissue to the nAVsos nerve at 6 mm SL. The nAVsos nerve (white arrowhead) now has lateral line tissue running parallel to it on the portions of the nerve that extend past the intersection point (white box) with the O2 and IO4 primordia (white brackets). (E) Proliferation of primordium tissue in the SO superficial line at 7 mm SL. The primordium extending from O2 (white bracket) still intersects (white box) with the SO superficial line, while the primordium from IO4 is beginning to condense into canal neuromast IO4. The first neuromast of the SO Superficial line (SO Sup 1) has begun to condense beyond the intersection point. (F) Differentiation of a supraorbital superficial neuromast by 8 mm SL. Both SO Sup 1 and canal neuromast IO4* have differentiated into mature neuromasts with functional hair cells (magenta), and neuromast O2 remains permanently connected to the SO superficial line (white bracket; white box). (F′) Same view as (F) showing the same specimen immunolabeled for neuromast marker Sox2 (magenta) and nerve marker acetylated alpha‐tubulin (cyan). The nAVsos nerve is labeled. (G–H) Representative images of SO supraorbital development in specimens labeled for Sox2 (magenta) and acetylated alpha‐tubulin (cyan) at (G) 5 mm, (H) 6 mm, (I) 7 mm, and (J) 8 mm SL. Annotations as in Panels C–F. Scale = 50 µm. (**K**) Schematic of the hybrid‐origin mechanism of SO superficial line formation at the imaged stages 5–8 mm SL with placodal origin indicated.

Unlike the primordium‐driven mode of development described above for the MD and IO superficial lines, there is no primordium tissue initially associated with the developing SO superficial nerve (nAVsos), which has emerged by 7 dpf (3 mm SL) (Figure 2A′). To investigate the origins of the neuromast‐producing LL tissue of the SO superficial line, we traced the development of the region immediately caudal to the eye between the 3 mm SL stage, when the nerve first appears, and the 8 mm SL stage, when the first SO superficial neuromast has formed over the dorsal‐most point of the orbit (Figure 5). Panel 5A is a low magnification view showing the AV‐derived presumptive canal neuromast HM1, as well as AD‐derived neuromasts IO4 and O2. We next visualized this region in high magnification confocal maximum projections, in living specimens, using the *Tg(CldnB:GFP;Hgn39d:GFP)* double transgenic, which labels both LL progenitor tissue and LL afferent nerves with GFP (yellow), together with TMRE vital dye to mark mature neuromasts (magenta). We additionally used anti‐Sox2 immunolabeling to clarify whether cells visualized with the *Tg(CldnB:GFP;Hgn39d:GFP)* double transgene marker are LL cells, nerves, or neither (see Section 2.4 for details). Figure 5B,B′ confirms that the Sox2‐positive nAVsos nerve does not have any LL primordium cell nuclei directly associated with it (magenta; red arrowheads), and that *CldnB:GFP*‐positive cells extending from existing neuromasts are interneuromast cells with Sox2‐positive nuclei (white arrowheads).

Figure 5C–F shows a time series of SO superficial LL formation in a representative single living specimen (one of *n* = 5). Figure 5C shows that at 5 mm SL, the newly forming nAVsos nerve extends dorsally (white arrowhead) past the level of neuromasts IO4 and O2. Each of these neuromasts extends a strand of *CldnB:GFP‐*labeled interneuromast cells (brackets) that meet to form a “bridge” of tissue, which the extending nerve intersects (intersection indicated by white box). A few days later, at 6 mm SL (Figure 5D), the nerve has extended beyond the bridge of tissue and up over the eye (out of frame in this panel). By this stage, additional primordium tissue is present along the nerve, at points beyond (i.e., dorsal to) the intersection with the tissue bridge (white arrowhead: Figure 5D). By the 7 mm SL stage (Figure 5E), the nAVsos nerve has extended fully, to reach a location above the dorsal‐most point of the orbit, and the cells along the nerve appear to be beginning to proliferate and condense into the first SO1 superficial neuromast. In addition, at this same stage, the tissue extending dorso‐anterior from IO4 loses contact with the SO superficial line, and this tissue now establishes a “bud” from IO4. This tissue goes on to form a new canal neuromast IO4* (Figure 5F). By 8 mm SL, the first SO superficial neuromast (SO Sup 1) has matured to become fully TMRE‐positive (Figure 5F). Staining this 8 mm SL endpoint for Sox2 (magenta) and the nerve marker acetylated alpha‐tubulin (cyan; Chitnis and Kuwada 1990) confirms that the tissue projecting from IO4 and O2 to the nAVsos nerve is composed of interneuromast cells (Figure 5F,F′), and that neuromast progenitor cells are not present along the nerve before its intersection with the AD‐derived neuromast progenitor cells.

Figure 5G–J shows representative confocal images of fixed specimens labeled with Sox2 (magenta) and acetylated alpha‐tubulin (cyan) antibodies, which trace the hybrid‐origin mechanism of SO superficial line development through a series of steps: (1) At 5 mm SL the naked nAVsos nerve extends (white arrowhead) to meet the tissue “bridge” made by the interneuromast cells of AD‐derived neuromasts IO4 and O2 (Figure 5G, brackets). (2) At 6 mm SL, the nerve “catches” interneuromast tissue, which is now seen running parallel to the nerve after the initial intersection point (Figure 5H, white box). (3) At 7 mm SL, the nerve and interneuromast tissue are migrating together up and over the eye towards their final position above the dorsal‐most point of the orbit (Figure 5J; white arrowhead). (4) Interneuromast cells expand in number (presumably by proliferation), with the first neuromast of the SO superficial line forming above the dorsal‐most point of the orbit (Figure 5J; SO Sup 1). Figure 5K is a schematic of the steps of the mechanism described in Figure 5G–J, with the origin of each neuromast labeled as AV‐derived (magenta) or AD‐derived (cyan). This novel, hybrid‐origin mechanism of neuromast formation is particularly significant because it combines a nerve that derived from the AV system with neuromast tissue derived from the AD system to form a new neuromast line without the use of a shared primordium. Of note, this is the latest of all the lines in the anterior LL system to form, with development of the first SO superficial neuromast completed at 8 mm SL. Additional superficial neuromasts will be added along this line throughout ontogeny.

Each of the mechanisms of larval stage neuromast formation generates characteristic innervation patterns. Thus, the adult innervation patterns allow us to infer how any given neuromast developed. For neuromasts formed by the new hybrid‐origin mechanism (Figure 5), or migrating primordia (Figure 3), a new branch extends from gAV or a gAV‐derived nerve. Because neuromasts that are innervated by a common nerve usually originate from the same primordium, daughter neuromasts that descend from founder neuromasts by budding will be directly connected to their founder neuromast by a new nerve branch (Figure 4D,I). Consistent with this observation, a previous analysis of the development of the opercular line showed that superficial neuromast budding creates a “pedigree pattern” (Wada et al. 2010). In contrast, we observe that neuromasts that form by intercalation between two existing neuromasts are innervated by a new nerve branch from the original, primary line (Figure 4H, J). Analyzing the innervation patterns of 22 mm SL adult specimens (Figure 2; Table 2) shows that superficial neuromasts are added primarily by budding and intercalation during larval stages, while the founder neuromasts of each superficial line are established by migrating primordia and the novel hybrid‐origin mechanism.

Table 2 integrates data from Figures 1–5 to summarize the mechanisms of neuromast formation for each canal neuromast and for each superficial line or series. In the SO line, canal neuromasts SO1‐3 are initially deposited during embryonic development, with neuromast SO1* budding from SO1 at 5 mm SL. In the IO line, canal neuromasts IO1, IO2, and IO4 form during embryonic development, with IO3 added shortly after in 4 dpf larvae, and three additional neuromasts forming during later larval stages, two by budding (IO2* and IO4*) and one by intercalation (IO3*). The MD line forms later, with only two MD canal neuromasts deposited by 3 mm SL (HM1 and HM2). By adult stages, another five MD neuromasts have formed by intercalation from the interneuromast progenitors along the MD canal. The first neuromasts of the superficial lines in general originate later—up to 8 mm SL in the case of the SO Superficial line—and each of the superficial lines continues to add neuromasts up to and during adulthood (> 22 mm SL). Our analysis reveals that a large proportion of both canal neuromasts and superficial line neuromasts originates during larval stages.

### 3.4 Innervation Is Necessary for Anterior LL Development

In the posterior LL, innervation is necessary for the formation of superficial neuromasts via the budding mechanism (Wada et al. 2013). However, the role of innervation in the development of the anterior LL is unknown. Based on the prominence of innervation in the newly described hybrid‐origin mechanism of SO superficial anterior LL formation, we hypothesized that innervation might be required for the development of this superficial line. In addition, we wished to evaluate whether innervation is necessary for the development of additional components of the system, such as neuromasts formed through intercalation, or for canal neuromast maturation.

**FIGURE 6:**
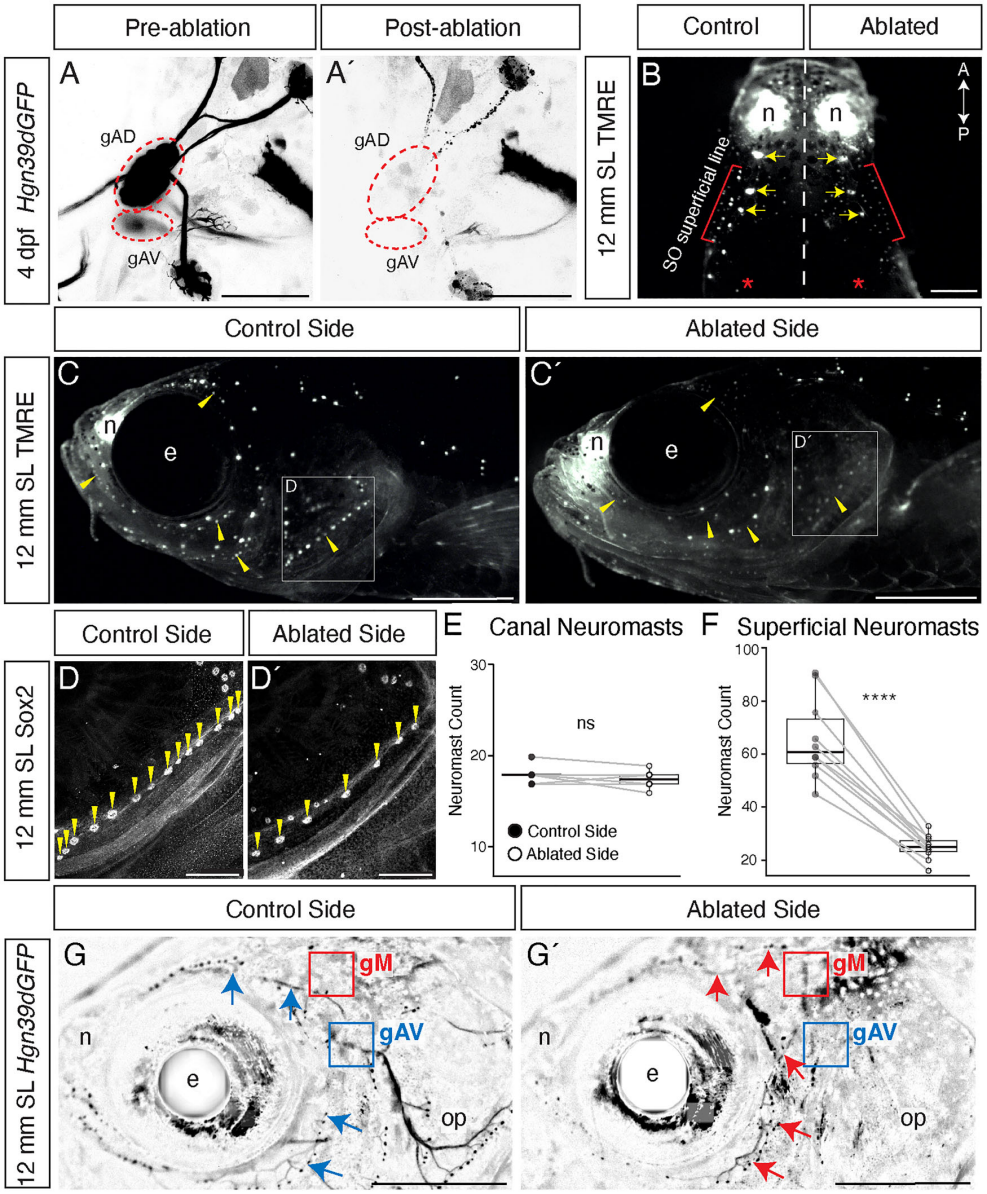
Superficial neuromast pattern and number is severely disrupted in the absence of innervation. (A–A′) Confocal images of the anterior LL anterodorsal (gAD) and anteroventral (gAV) ganglia labeled by *Tg(Hgn39d:GFP)* (yellow ovals) before (A) and immediately after (A′) laser ablation at 4 dpf. Scale = 50 µm. (B–D′) Images of a representative single 12 mm specimen that had undergone unilateral anterior LL ganglia ablation. (B) Dorsal view showing both control and ablated sides labeled with TMRE. The supraorbital superficial line (red bracket) is labeled parallel to the supraorbital canal line (yellow arrows) along with the anterior pit line (red asterisk). Scale = 1 mm. (C–C′) Lateral views showing control (C) and ablated (C′) sides. Yellow arrowheads indicate areas where superficial neuromast patterning was strongly reduced compared to the control side. The white boxes indicate the area magnified in (D–D′). Scale = 1 mm. (D–D′) Higher magnification confocal (10× objective) view of the opercular superficial neuromast line in the same specimen immunolabeled for Sox2, showing control (D) and ablated (D′) sides. Neuromasts are indicated with yellow arrowheads. Scale = 200 µm (E) Box plot showing change in canal neuromast number between the ablated side and internal unablated control side (*n* = 10). (F) Box plot showing change in superficial neuromast number between the ablated side and unablated control side (*n* = 10). Gray lines indicate paired values. A Shapiro–Wilk test was performed on all data to confirm normality before performing a paired t‐test. Not significant (ns) = *p* > 0.05; ****p < 0.0001. (G–G′) epifluorescent microscopy lateral views showing control (G) and ablated (G´) side nerves labeled by *Tg(Hgn39d:GFP)*. Red boxes indicate the middle ganglion (gM) of the posterior LL system of each side, and blue boxes indicate the anteroventral ganglion (gAV). (G) Blue arrowheads indicate nerve tracts from gAV (blue box) innervating supraorbital and infraorbital superficial neuromasts (black dots), while gM is not visible (red box). (G′) Red arrowheads indicate nerve tracts from gM (red box) ectopically innervating supraorbital and infraorbital superficial neuromasts, while gAV has been ablated (blue box). Scale = 1 mm. e, eye, n, nostril, op, operculum.

Figure 6 shows the neuromast patterns of *Tg(Hgn39d:GFP)* zebrafish specimens in which we performed a unilateral ablation of both the gAD and gAV ganglia at 4 dpf to permanently remove innervation of the anterior LL system on one side of the head. The experimental animals were raised to 6 weeks post fertilization (wpf), representing a range of stages between 11 and 15 mm SL. At these stages, all superficial lines have formed, and canal neuromasts now sit within ossifying canal grooves (Webb and Shirey 2003).

In successful ablations, the signal from both the gAD and gAV ganglia was completely and permanently removed, and the anterior LL neuromasts began to denervate immediately, as shown by confocal maximum projections in Figure 6A,A′ (*n* = 10). We found no evidence for regeneration of either the ganglia or their nerves following the ablations. For each experimental fish, the control and ablated sides were imaged with an epifluorescent microscope and assayed using the live dye TMRE (Figure 6B,C′), followed by clearing and staining for the neuromast marker Sox2 for a higher resolution confocal assay of the neuromast pattern (Figure 6D,D′). Figure 6B shows a dorsal view (anterior to the top) of the change in neuromast pattern in a single specimen between the control and the ablated side. While each of the SO canal neuromasts (yellow arrows) is present on the ablated side, the SO superficial line is completely absent (red brackets), and the anterior pit line (introduced as line 10 in Figure 1A) is missing neuromasts (red asterisks). Importantly, in 8/8 experimental specimens assayed, all the neuromasts that lie adjacent to the SO canal line were missing at 6 wpf, indicating that the SO superficial line did not form. This finding confirms that the novel hybrid‐origin mechanism that forms the SO superficial line does indeed require innervation.

Observing the crania of the specimens that had undergone ganglion ablation in lower magnification views, we found a dramatic reduction in the number of superficial neuromasts across the entire ablated side. Figure 6C compares lateral views of the control (Figure 6C) and experimental (Figure 6C′) sides of the same specimen shown in Figure 6B, with yellow arrowheads indicating locations where superficial neuromasts have been drastically reduced in response to ablation. Neuromasts on the ablated side also show a decrease in TMRE intensity compared to posterior LL neuromasts on the same side, or control neuromasts on the contralateral side, suggesting a decrease in the number of functional hair cells per neuromast (Figure 6B–C′). Close observation of the superficial neuromast pattern reveals that both the IO superficial and MD superficial lines are present at their full lengths, but they comprise fewer total neuromasts. Another example of reduction in superficial neuromast number is found in the opercular line, where in this specimen the control side (Figure 6D) has 14 neuromasts (yellow arrowheads), but the ablated side (Figure 6D′) has only 7 neuromasts (yellow arrowheads), which are more widely spaced. This observation is consistent with a previous description of opercular line development, in which the line forms through sequential budding but proliferates further as the animal grows and the original neuromasts move apart (Wada et al. 2010), indicating that innervation becomes required for this secondary phase of development.

Notably, quantification reveals that the total number of canal neuromasts is not significantly different in specimens that had undergone ablation relative to unablated or sham‐ablated controls, suggesting that innervation is not required for canal neuromast formation (Figure 6E). The average number of total neuromasts decreased significantly, from 83.8 to 42.3, with this reduction almost entirely explained by the average reduction in the number of superficial neuromasts from 65.7 to 24.9 (Figure 6F). Moreover, the phenotype is very consistent, with all specimens showing a significant decrease in superficial neuromast number and similar disruptions to their complex neuromast patterns. We conclude that innervation is required for a significant proportion of the larval‐stage formation of superficial neuromasts, regardless of the specific patterning mechanism used.

Our ganglia ablation experiments additionally reveal a capacity for the anterior LL nerves from one placode to promiscuously innervate nearby neuromasts derived from another placode. In almost all specimens where the ganglia were fully ablated (13/14), innervation crossed over from the control side to innervate the nasal line neuromasts on the ablated side. Moreover, in a small proportion of specimens that had undergone ganglia ablation (4/14), the middle ganglion of the posterior LL (gM) (Raible and Kruse 2000) ectopically reinnervated the anterior LL, with multiple nerve fibers contacting both superficial and canal neuromasts of the anterior LL system and partially rescuing the number and pattern of neuromasts. Figure 6G,G′ compares the unablated control side (Figure 6G) with the ablated side (Figure 6G′) of an experimental animal showing this phenotype. Figure 6G′ shows that a thick ectopic nerve tract extends ventrally from gM to reinnervate superficial neuromasts (red arrows) normally innervated by gAV, restoring their typical patterning (compare Figure 6G with Figure 6G′). This rescue capacity of the nerve fibers provides another example of innervation promoting superficial neuromast development.

**FIGURE 7:**
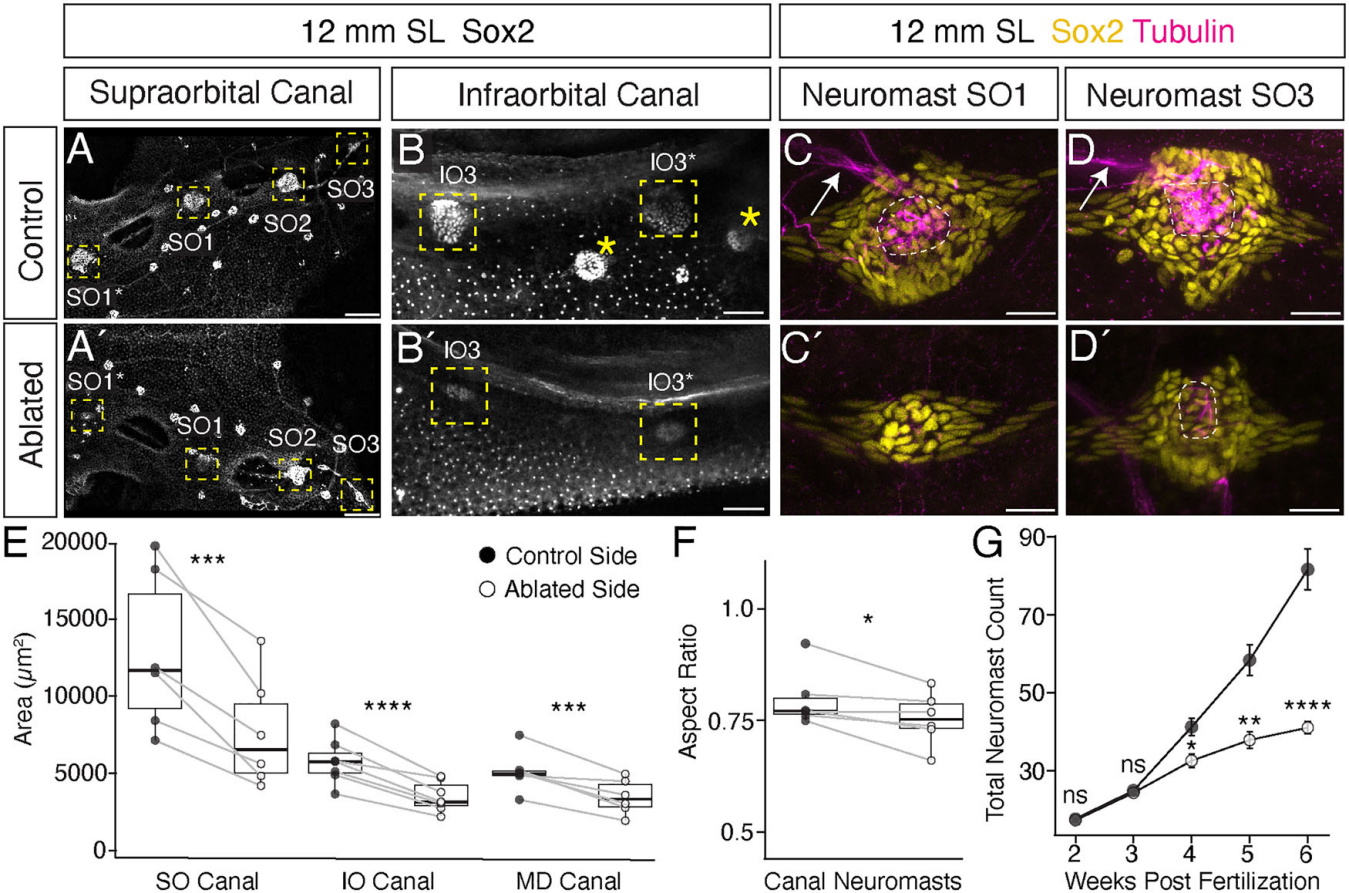
Loss of innervation disrupts canal neuromast size and morphology. (A–D′) Comparison of canal neuromasts on the control and ablated sides of a 12 mm specimen, immunolabeled with Sox2 (white, yellow) and acetylated alpha‐tubulin (magenta). (A, A′) dorsal view of canal neuromasts SO1*‐SO3 (yellow boxes). (B, B′) Lateral view of canal neuromasts IO2 and IO3 in the same specimen; yellow boxes indicate canal neuromasts, yellow asterisks indicate superficial neuromasts. Scale = 50 µm. (C, C′) Dorsal high magnification views showing morphology of canal neuromast SO1 on unablated control (C) versus ablated side (C′). (D, D′) Dorsal high magnification views showing morphology of neuromast SO3 on unablated control (D) versus ablated side (D′). Scale = 20 µm. (E‐F) Quantifications of canal neuromast area and aspect ratio. Gray lines indicate paired values. (E) Dot plot showing the difference in average canal neuromast area for the supraorbital (*n* = 6), infraorbital (*n* = 7), and mandibular *(n* = 6) canals in the control (solid circle) and ablated (open circle) conditions. (F) Dot plot (*n* = 6) showing the average canal neuromast aspect ratio for control and ablated conditions. (**G**) Total neuromasts versus weeks post fertilization on the control and ablated sides of specimens at 2 wpf (*n* = 6), 3 wpf (*n* = 6), 4 wpf (*n* = 6), 5 wpf (*n* = 6), and 6 wpf (*n* = 8). A Shapiro–Wilk test was performed on all data to confirm normality before performing a paired *t*‐test. *p < 0.05, **p < 0.01, ***p < 0.001, ****p < 0.0001.

While loss of innervation does not reduce the number of canal neuromasts, it does disrupt canal neuromast size and morphology, as shown in Figure 7. Figure 7A–D′ shows confocal maximum projection images of canal neuromasts on the control and ablated sides of the same specimen. Figure 7A, A′ compares a dorsal view of the SO canal on control and ablated sides in a 12 mm SL specimen, cleared and stained for Sox2. On the ablated side, neuromast SO3 is similar in size to the control side, and neuromast SO2 is slightly smaller than its counterpart. There is a much more striking difference for SO1 and SO1*, where the neuromasts on the ablated side are a fraction of the size on the control side, and express Sox2 at lower levels. Similar morphological changes can be observed in the IO and MD canals. For example, on the ablated side, IO3 and IO3* show a strongly reduced Sox2 signal, are closer to the size of superficial neuromasts, and are more elongated than the circular control side neuromasts (compare Figure 7B, B′).

To visualize changes in canal neuromast morphology in more detail, we also stained 11–15 mm SL specimens for acetylated alpha‐tubulin, which labels innervating nerves as well as the soma and kinocilia of sensory hair cells (López‐Schier et al. 2004) (Figure 7C–D′). On the control side, the overall shape of neuromast SO1 is round, with a large circle of sensory hair cells (magenta; white outline) located in the center of the support cells (yellow; Figure 7C). By contrast, the SO1 neuromast on the ablated side is significantly smaller and more elongated; it appears to have lost its sensory hair cells and is composed entirely of Sox2‐expressing support cells (yellow; Figure 7C′). Canal neuromast SO3 on the control side has very similar morphology to SO1 (Figure 7D), while its counterpart on the ablated side is slightly smaller, has fewer Sox2‐expressing cells (yellow), and appears to have fewer hair cells (magenta; white outline) (Figure 7D′).

During regeneration, it has been shown that Sox2 is necessary for both the turnover of neuromast support cells and the differentiation of hair cells from progenitors (Hernández et al. 2007). In neuromasts lacking innervation, we observe significantly fewer Sox2‐expressing cells (Table 3), and reduced levels of Sox2 expression, as well as reduced canal neuromast size and fewer hair cells, as revealed by a decrease in both tubulin and TMRE signal. These findings are consistent with a potential role for Sox2 in developing canal neuromast support cell proliferation and hair cell differentiation. While canal neuromast morphology is altered in the absence of innervation, the canals themselves appear to be properly induced and patterned. In the posterior LL, neuromasts are required for canal formation (Wada et al. 2014); by contrast, the presence of normal canals in the cranial region of our manipulated specimens suggests that loss of innervation, and resultant changes in neuromast morphology, do not disrupt cranial canal ontogeny.

**TABLE 3:** Loss of innervation reduces the size of canal neuromasts.

	Average number of Sox2+ neuromast support cells		
	Control Side	Ablated Side	*p* value
Supraorbital Line	67.9 ± 10.1	49.3 ± 9.1	0.0042
Infraorbital Line	41.2 ± 4.3	27.6 ± 1.6	0.0015

*Note:* We compared the size of presumptive canal neuromasts between the unablated control side and the ganglia‐ablated side of experimental specimens (*n* = 6) by counting the number of Sox2‐expressing support cells in the neuromasts. Average counts are provided for the Sox2+ cells in all neuromasts of the presumptive supraorbital line and the infraorbital line. Counts were performed on confocal *z*‐stacks (see Figure 7 for examples).

To quantify changes in canal neuromast size and shape, we calculated the area of each canal neuromast in control versus experimental specimens (*n* ≥ 6) by measuring neuromast length (parallel to the canal) and width (perpendicular to the canal). In all three canals, the area of the canal neuromasts was reduced significantly in the ablated condition (Figure 7E). In zebrafish, canal neuromast maturity is described by the increasing width of the canal neuromasts with growth (Webb and Shirey 2003), such that the neuromast aspect (width/length) ratio increases as ontogeny proceeds. Comparison of the aspect ratio between the control versus experimental specimens (*n* = 6) reveals a significantly lower ratio on the ablated side, suggesting that these canal neuromasts show arrested growth compared to their innervated counterparts (Figure 7F). These results show that while innervation is not required for canal neuromast formation or canal induction, it does become required for proper canal neuromast morphology and growth.

Building on the consistent disruption of superficial neuromast pattern and decrease in canal neuromast size of the ablation phenotype, we sought to identify the zebrafish age at which a developmental switch occurs, after which innervation becomes required for anterior LL patterning. Specimens that had undergone the ablation procedure at 4 dpf were incubated in TMRE once each week to assay neuromast counts on both the control and ablated sides (Figure 7G). At 2 wpf (4 mm SL; *n* = 6) and 3 wpf (5–6 mm SL; *n* = 6), the number of neuromasts is nearly identical between the control and ablated conditions, showing that innervation is not required for anterior LL development during early larval stages. There are only small standard deviations in the overall neuromast counts at these stages, in line with the data collected from unmanipulated controls (compare with Figure 1F). However, by 4 wpf (7–8 mm SL; *n* = 6), the number of neuromasts on the control side is significantly higher, and we observe noticeable differences in patterning, for example, the first neuromasts of the SO superficial line failing to form. After this stage, the number of neuromasts on the ablated side plateaus, while neuromasts continue to be added on the control side. This quantification reveals a sharp developmental switch at 4 weeks post‐fertilization (7–8 mm SL), at which stage innervation becomes required for further superficial neuromast formation by any mechanism.

In summary, our results have established that innervation is required for the addition of superficial neuromasts by any mechanism, including the hybrid‐origin development of the SO superficial line, as well as the growth and morphology of canal neuromasts, after approximately 7 mm SL.

## 4 Discussion

Here, we provide the first description of the patterning of the zebrafish anterior LL sensory neuromasts and their innervation at stages between 10 dpf and adulthood. We find that the anterior LL system undergoes significant secondary development during larval stages, when three major lines of presumptive superficial neuromasts are established that run parallel to existing lines of presumptive canal neuromasts. By 22 mm SL, when the fish are considered adult, the system has expanded dramatically, in large part due to the development of three gAV‐innervated lines of superficial neuromasts that run parallel to the three existing lines of canal neuromasts. These superficial neuromasts are added through the migration of new primordia, intercalation, budding, and by our newly described hybrid‐origin mechanism (summarized in Table 2). At around 4 wpf/7 mm SL, innervation becomes required for neuromast formation: when the anterior LL ganglia are ablated, almost no superficial line expansion occurs after 4 wpf/7 mm SL, but the underlying dermal bones continue to expand, leading to increasingly sparse neuromast distribution. In addition, innervation is necessary for proper canal neuromast growth and morphology. Importantly, the peak in neuromast addition rates at late larval stages, seen in normal ontogeny, coincides with the developmental switch to innervation dependence, showing that innervation drives a distinct secondary phase of anterior LL development.

In the zebrafish posterior LL, all lines of presumptive canal neuromasts and presumptive superficial neuromasts have originated by the end of embryonic development at 3 dpf (Ledent 2002). By contrast, the cranial anterior LL generates half of its canal neuromasts and all its major lines of superficial neuromasts at larval stages, showing both temporal and mechanistic differences from the development of the comparatively simpler trunk posterior LL. This difference correlates with the requirement that the complex cranial anterior LL system must navigate around a variety of morphological features, unlike the obstruction‐free structure of the trunk.

We find that the anterior LL neuromasts formed during larval and juvenile stages, following initial establishment of lines of superficial neuromasts, are added through the continued use of embryonic budding and intercalation mechanisms—mechanisms that are also used in the expansion of the posterior LL. During this process, both superficial and canal neuromasts are added to the anterior LL system as the fish grows. However, it is unclear how neuromasts acquire “canal” versus “superficial” identity. The superficial line neuromasts can only produce more superficial neuromasts. By contrast, when canal neuromasts bud, they can produce daughter neuromasts of either canal or superficial identity. Moreover, the identity of these daughter neuromasts does not appear to correlate with either the time or the general location of their formation. For example, at 6 mm SL, presumptive canal neuromast IO1 buds off superficial neuromast daughters, while adjacent presumptive canal neuromast IO2 buds to form presumptive canal neuromast daughter IO2*. Despite their proximity, these anterior LL neuromasts are spatially distributed, such that differential signaling from the local tissue environment or adjacent neuromasts might regulate daughter fate. In a related project, we have established that cranial neural crest cells are required for aspects of early anterior LL patterning, providing an example of how a local tissue environment can influence neuromast development (Venkataraman, McGrory, et al. 2025).

In addition to the previously described mechanisms of superficial neuromast formation, we have uncovered a novel mechanism that drives the development of the SO superficial line of neuromasts. In this hybrid‐origin mechanism, a nerve from the AV system interacts with interneuromast cells extending from neuromasts that are innervated by the AD system, and the extending nerve and primordial neuromast cells then migrate together dorsally, towards the dorsal‐most point of the orbit. Moreover, using a ganglion ablation strategy, we have demonstrated that this nerve is required for the migration and apparent proliferation of these interneuromast cells. This mechanism is unique because it generates a superficial LL in which neuromast progenitor tissue originates from the inferred AD placode, whereas the nerve that innervates those neuromasts originates from the inferred AV placode. In all other described mechanisms, the nerve associates closely with the tissue of the relevant anterior LL primordium immediately after its formation, such that these other lines have a consistent placodal origin (Wada et al. 2010 and this study).

Paralleling the formation of neuromasts by the hybrid‐origin mechanism, we have observed that in experimental situations, the anterior LL nerves from one placode have the capacity to promiscuously innervate nearby neuromasts from another line or placode. For example, in our ablation experiments, we found that innervation frequently crossed over from the control side to innervate the nasal line neuromasts on the ablated side. On a few occasions, the middle ganglion of the posterior LL ectopically reinnervated the anterior LL to partially rescue neuromast patterning. This latter observation supports a model in which innervation can induce the development of superficial neuromasts after interacting with proximal interneuromast progenitor tissue through an inefficient neurotrophic effect. We speculate that the more developmental mechanisms the LL system has in its “toolbox,” the more permutations of patterning are possible, suggesting mechanistic underpinnings for the remarkable diversity of LL patterns across teleost species (Pichon and Ghysen 2004; Wada et al. 2008; Webb 1989).

While this study has uncovered differences in the development of the anterior and posterior LL systems, it has also revealed commonalities. Both the zebrafish anterior LL (this study) and posterior LL (Wada et al. 2013) show innervation‐dependent budding of superficial neuromasts at late developmental stages. For example, ablation of the posterior LL ganglion blocks the formation of posterior LL superficial neuromast clusters that form by the budding mechanism from an embryonic founder neuromast, beginning at approximately 10 mm SL (Wada et al. 2013). Moreover, in these posterior LL ganglion ablated specimens, the founder neuromast shows an initial extension of tissue, suggesting that innervation is required only for the later steps of proliferation and differentiation in the budding process. In addition to budding, we have established that innervation is required for other mechanisms of superficial neuromast patterning in the anterior LL. Specifically, superficial neuromasts that are added by the intercalation mechanism no longer form after approximately 7 mm SL in specimens with ablated ganglia. The SO line of superficial neuromasts, which we have demonstrated forms via a previously undescribed hybrid‐origin mechanism, also fails to form in the absence of innervation. Although we have not directly demonstrated cell proliferation during the development of the superficial neuromasts, the significant increase in tissue that we observe strongly suggests that it must be occurring. Assuming that to be the case, understanding how the nerve modulates the proliferation of LL tissue at a molecular level will be essential to gain further understanding of this potentially instructive nerve‐organ relationship.

Previous work suggests that Wnt/beta‐catenin signaling is a prime candidate to direct neuromast expansion. Wnt/beta‐catenin signaling in the leading edge of migrating posterior LL primordia is the driving force of primordial proliferation and neuromast formation (reviewed by Dalle Nogare and Chitnis 2017). At larval stages, the Wnt effector protein Lef1 is expressed in proliferating cells during neuromast budding, and this expression is strongly reduced in the absence of innervation (Wada et al. 2013). The underlying mechanism that nerves use to induce superficial neuromast proliferation may thus be direct local activation of Wnt signaling. In support of such a model, Iwasaki et al. (2020) have shown that nerve‐derived Rspo2/Wnt signaling is required for proliferation and subsequent budding of some posterior LL superficial neuromasts, as well as for embryonic development of HM1/two neuromasts.

We have established that innervation is also required for the proper growth of anterior LL canal neuromasts. Reporter lines for the Wnt effector protein Lef1 indicate that canal neuromasts strongly upregulate Wnt signaling during canal formation (13–15 mm SL), while the signal is undetectable in mature superficial neuromasts, suggesting that continuous Wnt expression promotes canal neuromast growth (Wada et al. 2014). We also observed that both the size of canal neuromasts and the number of hair cells decreased in the absence of innervation, which is consistent with the decrease in hair cells observed in denervated superficial neuromasts of the posterior LL (Wada et al. 2013). We suggest that future studies should investigate whether nerves in the anterior LL produce a ligand that induces Wnt signaling in both superficial and canal neuromasts.

The innervation dependence of LL system expansion that we have uncovered represents a striking reversal of the typical “organ‐first” relationship observed in sensory system development. Generally, nerves are dispensable for development, with sense organs differentiating from their placodes prior to innervation. For example, in the mammalian inner ear, sensory tissue develops autonomously and later attracts axons through neurotrophic factor secretion (Fritzsch et al. 1997). Similarly, during embryonic posterior LL development, the primordium produces glial cell line‐derived neurotrophic factor (GDNF), which is required for the extension of GDNF receptor‐expressing nerves (Thomas et al. 2015). Our findings have revealed that the anterior LL undergoes a novel developmental switch from sensory tissue guiding innervation to the requirement of innervation for superficial neuromast development.

What might be the functional purpose of this developmental switch for aquatic vertebrates? First, nerves may represent an efficient mechanism to drive cell proliferation as body size increases. Rather than relying on collective primordium migration over increasingly large distances, a single neuron already innervating a neuromast could locally release signaling molecules to stimulate the cell proliferation needed to produce additional superficial neuromasts. Second, a two‐step system could allow the LL to perform adaptive functions across life stages. For rapidly developing aquatic vertebrates, fast, primordium‐driven early development ensures that hatchlings have the sensory function needed for survival. As ontogeny proceeds, slower, nerve‐dependent cell proliferation may permit diversification of adult neuromast patterns according to species‐specific sensory needs (Wada et al. 2013). Consistent with this idea, the switch to nerve‐dependent superficial neuromast development correlates with the onset of metamorphosis at the larva‐to‐juvenile transition (McMenamin and Parichy 2013).

While we have provided a detailed account of the late patterning of the anterior LL in zebrafish, comparative studies will be required to address how diverse neuromast patterns are established across species. Our review of previous studies documenting LL innervation patterns reveals that the expanded gAV innervation system that we have described for zebrafish is a common teleost feature (Asaoka et al. 2012; Asaoka et al. 2014; Münz 1979; Nakae et al. 2006; Nakae et al. 2021; Nakae and Sasaki 2010; Northcutt et al. 2000). However, a gAV‐innervated SO superficial line has not been previously documented, raising the question of whether LLs form with hybrid origins in other species. Fortunately, the whole‐mount clearing and immunolabeling methods that we have described in this work are applicable to a variety of aquatic vertebrate species. Ultimately, comparisons of not only adult neuromast patterns, but also innervation patterns and developmental mechanisms, will clarify which aspects of late anterior LL development are zebrafish novelties, versus which aspects are conserved features of patterning systems fundamental to fishes.

## Author Contributions

**Theresa J. Christiansen**: conceptualization and study design, development of methodology, experimentation and imaging, data analysis and interpretation, writing – original draft, writing – review and editing. **Vishruth Venkataraman**: conceptualization and study design, development of methodology, experimentation and imaging, data interpretation, supervision, writing – review and editing. **Victoria E. Prince**: data interpretation, overall supervision of the project, writing – original draft, writing – review and editing, funding acquisition, resources.

## Conflicts of Interest

The authors declare no conflicts of interest.

## Data Availability

The authors have nothing to report.
